# Machine-Milking Practices, Animal Welfare-Related Reactions and Quality of Milk Produced in Dairy Sheep Farms

**DOI:** 10.3390/ani15213078

**Published:** 2025-10-23

**Authors:** Dimitra V. Liagka, George C. Fthenakis, Stella N. Kalonaki, Konstantina S. Dimoveli, Daphne T. Lianou, Vasia S. Mavrogianni, Charalambia K. Michael, Mariangela Caroprese, Vassiliki Spyrou, Natalia G. C. Vasileiou

**Affiliations:** 1 Faculty of Animal Science, University of Thessaly, 41110 Larissa, Greece; 2 Veterinary Faculty, University of Thessaly, 43100 Karditsa, Greece; 3 School of Veterinary Medicine, European University of Cyprus, Engomi, 2404 Nicosia, Cyprus; 4 Department of Agriculture, Food, Natural Resources and Engineering (DAFNE), University of Foggia, 71122 Foggia, Italy

**Keywords:** animal-based indicators, dairy sheep, machine-milking, milk quality, somatic cell counts, total bacterial counts, welfare

## Abstract

**Simple Summary:**

The study aimed to evaluate potential issues regarding animal welfare, which may occur during the machine-milking process in dairy ewes, as well as the potential effects on the milk produced on these farms. The findings revealed a variety of reactions displayed by ewes during milking; these reactions were mainly related to increased movements of the legs of the animals. The findings suggest that incorrect practices during machine-milking caused nervousness and agitation to ewes, which indicates that these incorrect practices may potentially adversely affect their welfare; for example, prolonged duration of the milking process aggravated the reactions of ewes. Moreover, the somatic cell counts of the bulk-tank milk produced on the farms were found to correlate with the increased frequency of these reactions. The results indicate that handling of animals during milking should be carried out carefully and within a short time period, which will contribute to a better quality of the milk produced by these animals. The findings also underline that maintenance of animal welfare is linked to high product quality.

**Abstract:**

There is a scarcity of relevant information in the international literature regarding the welfare of dairy ewes during the milking process and the potential impact on the quality of the produced milk. Hence, there is a scope to further study potential interactions between the milking process and animal welfare in dairy sheep flocks. The specific objectives of this study were (i) the evaluation of the frequency of reactions potentially associated with the reduced welfare of dairy sheep during the milking process, (ii) the identification of predictors associated with the presence of these reactions, and (iii) the evaluation of associations with the quality of milk produced on a farm. The study was conducted in 52 dairy sheep farms in Greece. The farms were visited, and the milking process was observed and monitored, and the reactions of ewes during the milking process were recorded; samples from the bulk-tank milk were collected for somatic cell and total bacterial counting. Univariable and multivariable analyses were performed. The median duration of milking per row was 7.9 min, and the median duration of the milking process was 105 min. The reaction observed less often from ewes was vocalisation (in 9.6% of farms), and the one observed more frequently was ‘spot stepping’ (in 98.1% of farms). In total, eight different predictors were identified for the displaying of the various reactions observed and recorded; of these, two were related to the milking parlour and six were related to the milking process. Moreover, three different predictors were identified for the milk quality parameters; of these, one was related to the milking parlour and two were related to the milking process. Finally, a mild correlation was seen between the proportion of ewes that attempted to remove the milking cluster, and the somatic cell counts in the bulk-tank milk (*p* = 0.023). The results confirmed that incorrect practices during machine-milking can be stress factors for dairy sheep. In light of the present findings, procedures by milkers should be carefully carried out, which will contribute to minimising the nervousness and aggravation of ewes during milking. Stress factors can adversely affect the quality of milk produced on the farm. The findings underline that maintenance of animal welfare is linked to high product quality. This connection can further extend to consumer perceptions: the ethical treatment of dairy sheep is considered as an important facet of the overall food quality and sustainable production.

## 1. Introduction

The study of farm animal welfare has gained increased interest, driven by ethical considerations and economic benefits. Farm animal welfare is determined by interactions between animals and its environment (which includes a variety of determinants, e.g., people, other animals, farm infrastructure, climatological conditions) and focuses on addressing the needs of animals on a farm to ensure optimal living conditions [1,2]. Understanding stress responses and applying the respective knowledge to housing, husbandry, and human–animal interactions safeguards animal health and welfare and also improves production efficiency and product quality [3,4,5]. In general, a variety of physical or behavioural responses that can indicate frustration or inadequate environmental enrichment and, thus, reduced welfare levels, has been observed; such responses include avoidance of human contact, social isolation from the flock, increased lying periods, reduced rumination, and reduced feed and water intake [6,7,8].

In dairy animals, machine milking is a particularly important and labour-intensive farm action. During milking, animals are exposed to a multitude of physical and psychological stressors. For example, the milking system noise and/or the frequent close contact with people in the milking parlour are considered to potentially act as sources of stress for dairy animals [9,10,11,12]. In dairy cows, defaecation, urination and/or removing teatcups have been described as indicators of agitation and fear during the milking process. Moreover, a higher frequency of stepping and a significant reduction in vagal tone were observed during the period following the removal of the last teatcup to the release of animals compared to the period of udder preparation or the actual milking stage [13]. Stepping and kicking by animals has been described to be associated with restlessness and aggressiveness, respectively [11,14,15,16,17]. The presence of potentially stressing factors during the milking process may influence oxytocin release and thus lead to differences in milk yield [18,19].

There is a scarcity of relevant information in the international literature regarding the welfare of dairy ewes during the milking process and the potential impact on the quality of the produced milk. In a topic search in the Web of Science platform under the string {[stress OR welfare OR reaction] AND [sheep OR ovine OR *Ovis aries*] AND [milking] AND [process OR procedure]}, 182 articles were returned; however, a detailed examination of all these articles revealed that only the paper by Carcangiu et al. [20] dealt specifically with the welfare of sheep during the milking process. These authors reported that the characteristics of the milking parlour (type of entrance and room dimensions) presented stressful stimuli to ewes being milked, which adversely influenced the welfare of dairy ewes [20]. Hence, there is a scope to further study potential interactions between the milking process and animal welfare in dairy sheep flocks. The specific objectives of this study were (i) the evaluation of the frequency of reactions potentially associated with reduced welfare of dairy sheep during the milking process, (ii) the identification of predictors associated with the presence of these reactions and (iii) the evaluation of associations with the quality of milk produced on a farm.

## 2. Materials and Methods

### 2.1. Farms

The study was performed in dairy sheep farms in Central Greece, from November 2024 to May 2025, i.e., during the same milk collection season in the country. All farms were located in the region of Thessaly, within a distance of 75 km from the premises of the University of Thessaly. Farms were selected in a convenience mode, based on the willingness of farmers to accept a visit by university personnel for attendance during a milking session at the farm. Farmers were contacted by telephone; initial enquiries and discussions were made to explain the procedures and to confirm that inclusion criteria were met. In total, 54 farmers were contacted, but 2 of them (3.7%) did not show interest in a visit.

The criteria for the inclusion of farms into the study, set a priori, were as follows: (a) conventional (i.e., not organic) dairy sheep farm, (b) application of intensive or semi-intensive management system on the farm [21], (c) at least 100 ewes on the farm, (d) the application of machine-milking of ewes on the farm with a practice of two milking sessions daily.

Upon agreeing to a mutually convenient date for the visit, attendance during the morning or the evening milking session was decided at the toss of a coin. Finally, visits to farms were carried out on 17 morning and 35 evening milking sessions.

### 2.2. Details of Visits

Collection of information regarding the farm, relevant to the present study, was carried out by the investigators during the on-site visit, by means of a structured questionnaire (Table S1). This had been created specifically for this work and was based on a previously standardised questionnaire [22].

On arrival at each farm, after introductions, a brief interview of the farmer was performed. All the interviews were conducted by the same investigator (author D.V.L.). Each interview was performed with a member of the family that owned each farm, who managed and followed up on the business activities and operations in the sheep flock (‘farmer’). Additionally, as part of the information collected during the visit, details regarding the queries on the questionnaire were obtained and recorded.

Thereafter, during the milking session, the investigators performed observations and measurements and made appropriate notes regarding the milking process, remaining aside from the main action within the milking parlour and without interfering with the process (Table S1). The temperature and humidity within the milking parlour were recorded by means of a portable digital thermometer—hygrometer (Nahita; Auxilab, Beriáin, Navarra, Spain); three measurements were obtained, one 10 min after the start of the milking session, the second one hour later and the third at the end of the milking session (at which time milk samples were also collected from the cooling tank of the farm).

During the milking process, stopwatches available on the mobile telephones (iPhone 13; Apple Inc., Cupertino, CA, USA) of the investigators were used to record time points of various events that took place during the milking session. Specifically, five time-events were recorded during each milking row (Table 1), in order to subsequently calculate time intervals related to the milking process.

Moreover, the reactions of ewes during the milking process were also recorded. Seven different reactions were monitored and recorded. These are described in detail in Table 2. These reactions were recorded at an individual animal level (yes/no for each animal).

### 2.3. Sample Collection and Examination

Half an hour after completion of the milking session, samples were collected from the milk cooling tank (‘bulk-tank’) of the farm. During that half hour, the milk within the tank was being mixed thoroughly by the agitators present in the lid of the tank, which stirred the milk continuously until the cover (the lid) of the tank was raised for sample collection. The samples were collected directly from the bulk-milk within the cooling tank on each farm. The milk samples were collected by means of sterile plastic single-use pipettes; a pipette was immersed in the tank to withdraw a sample. In total, four 20 mL samples were collected from the milk cooling tank of each farm, and a new pipette was used for each sample. Immediately after collection, the milk samples were transferred into sterile plastic Universal-type vials.

Samples were stored at 0.0 to 4.0 °C using ice packs in portable refrigerators. They were immediately transported to the laboratory by car.

Of the four samples collected from the cooling tank, two were used for somatic cell counting, which was performed within two hours after sample collection, as detailed before [23]. The other two samples were used for total bacterial counting, which was performed by means of the technique described in detail by Laird et al. [24]. In all cases, duplicate tests were performed, i.e., each sample was divided in two sub-samples, which were tested separately.

### 2.4. Data Management and Analysis

All data were systematically recorded and organised using Microsoft Excel (Microsoft Corporation, Redmond, WA, USA). Data were analysed using SPSS v. 21 (IBM Analytics, Armonk, NY, USA).

The time intervals described in Table 3 were calculated based on the time-events recorded during the monitoring of the milking session. For the time intervals ‘duration of animal entrance into milking pens in each milking row’, ‘duration of milking of first ewe in each milking row’, ‘duration of milking process in each milking row’, and ‘duration of post-milking actions in each milking row’, the overall value for a farm was calculated as the median value of the values obtained for each milking row.

The following eight outcomes were considered: (i) proportion (%) of ewes that displayed ‘kneeling’ before entry to the milking pen, (ii) % of ewes that displayed ‘kneeling’ within the milking pen, (iii) % of ewes that urinated, (iv) % of ewes that defaecated, (v) display of vocalisation by ewes, (vi) % of ewes that displayed a kick-like reaction, (vii) % of ewes that attempted to remove the milking cluster (or removed it), and (viii) % of ewes that showed spot-stepping. These outcomes were calculated as the proportion of ewes that displayed the reaction of interest during the milking process, among all ewes milked on that session on the same farm, which, in the present study, was defined as the experimental unit. Moreover, the following ninth outcome was also considered: (ix) total number of distinct reactions observed from ewes on a farm during the milking session. This outcome was calculated as the total number of each of the above eight reactions (i–viii) observed during the milking process, monitored among all ewes that were milked on the farm on that session (possible values: 0 to 8).

A total of 48 independent variables were used to assess potential associations with each of the above outcomes (Table S2). These variables were related to general information about the farm and the farmer (*n* = 6), information about the milking parlour (*n* = 16) and information about the milking process (*n* = 26). Initially, univariable evaluations were performed between the independent variables (*n* = 48) and the outcomes (*n* = 8). Then, a multivariable model was developed for each of the above eight outcomes; those among the 48 independent variables found with *p* < 0.20 in the preceding univariable analyses were fed to the model. Then, variables fed into the model were progressively removed from the model using backwards elimination. The likelihood ratio test was performed to assess the *p* value of each independent variable; among those found with *p* > 0.20 [25], the one with the largest *p* was removed from the model. The procedure was repeated until no variable with *p* > 0.20 could be removed from the model [25]. The likelihood ratio test was employed to assess whether the removal of a variable led to a significant reduction in model fit. The variables included in the final assessment in each multivariable model are presented in Table S3. The associations between the variables that were used in the multivariable analysis (*n* = 9) for the proportion of ewes that attempted to remove the milking cluster (or removed it) were evaluated using a principal component analysis.

The farms were separated into three cohorts: cohort A included farms in which the proportion of ewes that displayed kick-like reaction or spot-stepping and of those that attempted to remove the milking cluster (or removed it) was below 4%. Cohort B included farms in which the proportion of ewes that displayed kick-like reaction or spot-stepping was over 4%, but the proportion of ewes that attempted to remove the milking cluster (or removed it) was below 4%. Cohort C included farms in which the proportion of ewes that displayed a kick-like reaction or spot-stepping and of those that attempted to remove the milking cluster (or removed it) was over 4%. The 4% threshold for dividing the farms was selected, because for the variable ‘Attempting to remove the milking cluster (or removing it)’, it represented the mean value among all farms in the study plus one standard deviation. The duration of the various time intervals calculated was compared between the three cohorts by using the Kruskal–Wallis test.

For all statistical analyses, somatic cell counts were transformed to somatic cell scores (SCS) as described by Wiggans and Shook [26] and Franzoi et al. [27]: SCS = log_2_ (SCC/100) + 3, whilst total bacterial counts were transformed to log_10_; for both parameters, the transformed data were used in the analyses. Then, for the presentation of the results, the transformed findings were back-transformed into 100 × 2^(SCS−3)^ and 10^log^ data, respectively.

Thereafter, the following two outcomes were considered: (i) somatic cell counts in the bulk-tank milk and (ii) total bacterial counts in the bulk-tank milk. Univariable analyses with the above 48 independent variables were performed initially. These were followed by multivariable analyses, by means of the same principle and methodological approach as detailed hereabove. The variables included in the final assessment in each multivariable model are presented in Table S4. After the completion of the multivariable analysis, analysis was carried out between the variables included in the final models for both the somatic cell counts and the total bacterial counts, after the allocation of the farms into clusters (Table S4). For the somatic cell counts, by using the relevant variables (*n* = 3; facilities for milk yield measurement, congestion of ewes before entry into the milking parlour, repeat milking of ewes), five clusters of farms were created through the combination of the findings in these variables. For the total bacterial counts, by using the variable ‘Number of milking units per animal milking position in the parlour’, four clusters were created, as follows: cluster 1 included farms with a ratio <0.5, cluster 2 included farms with a ratio equal to 0.5, cluster 3 included farms with a ratio between 0.5 and 1.0, and cluster 4 included farms with a ratio equal to 1.0 (the variable ‘number of milking units per animal milking position in the parlour’ reflects the number of animal positions served by one milking cluster).

Finally, each of the nine outcomes above was correlated with the results of somatic cell counts and total bacterial counts in the bulk-tank milk of the farm obtained from the farms. Spearman’s rank correlation analysis was used for this evaluation.

Statistical significance was defined at *p* < 0.05.

## 3. Results

### 3.1. Descriptive Results

The results of the observations performed during the visit, with regard to the milking parlour and the milking process are in Table S5.

#### 3.1.1. Time Intervals

**Figure 1:**
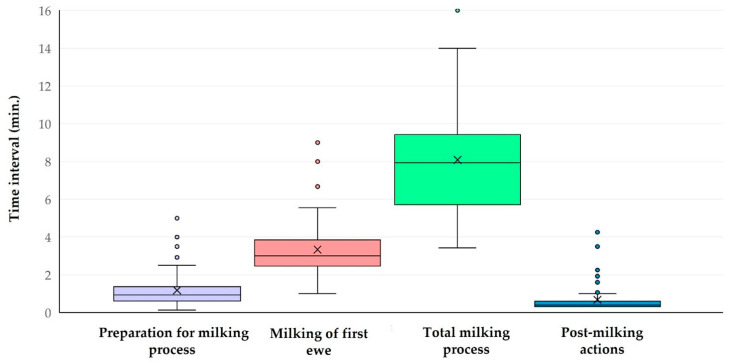
Box-and-whisker plot of four time intervals calculated for a milking row in dairy sheep farms.

**Figure 2:**
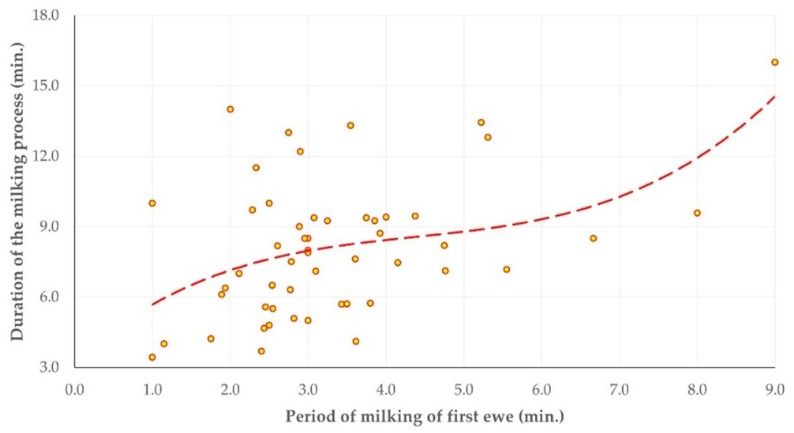
Scatter plot of the period of milking the first ewe versus the total duration needed for the milking process in a milking row (dashed line denotes the trend).

The results of the various time intervals calculated during the milking process are presented in Table 4 and Figure 1. Within the same milking row, there was a clear correlation between the period of milking of first ewe and the duration of the milking process (Spearman’s rank correlation, *r_sp_* = 0.352, *p* = 0.010) (Figure 2); there was also an inverse correlation between the duration of the milking process and the duration of post-milking actions (Spearman’s rank correlation, *r_sp_* = −0.343, *p* = 0.013).

#### 3.1.2. Animal Reactions

**Figure 3:**
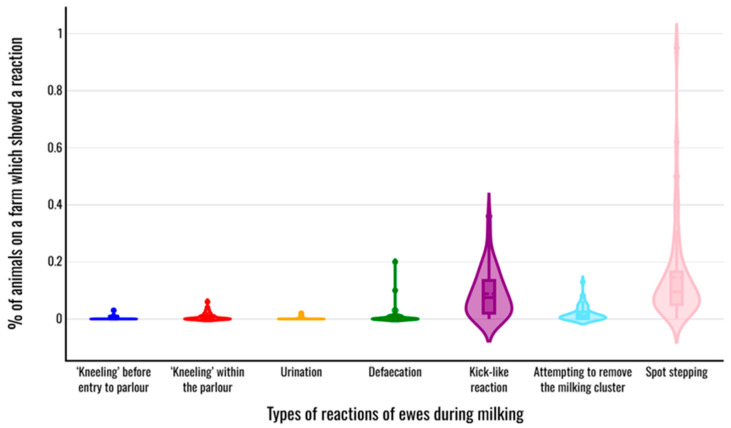
Violin plot of the within-farm prevalence of reactions in ewes during the milking process.

Each of the reactions in animals on the farms studied (Table 2) was observed and recorded in at least one farm of those studied. A reaction seen less often among the farms was vocalisation (observed in 5 farms (9.6% of all farms)), while the reaction seen more often was ‘spot stepping’ (observed in 51 farms (98.1% of all farms)) (Table 5, Figure 3). The median value of the total number of distinct reactions observed in all animals on a farm was 4 (interquartile range: 2).

The median value of within-farm prevalence of reactions among animals during the milking process varied from 0.0% for ‘kneeling’ before entry to the milking pen, ‘kneeling’ within the milking pen, and urination and defaecation, to 9.5% for displaying spot-stepping (Table 5). There was one farm in which no reaction was observed in any ewe during the monitored milking process and two farms in which seven of the eight reactions were observed in at least one ewe during the monitored milking process.

#### 3.1.3. Somatic Cell Counts and Total Bacterial Counts

Median value of somatic cell counts in the bulk-tank milk of the farms was 0.669 × 10^6^ (interquartile range: 0.521 × 10^6^) cells mL^−1^. Median value of total bacterial counts in the bulk-tank milk of the farms was 48 × 10^3^ (interquartile range: 127 × 10^3^) colony-forming units mL^−1^. There was a tendency for correlation between the somatic cell counts and the total bacterial counts in bulk-tank milk (Spearman’s rank correlation, *r_sp_* = 0.241, *p* = 0.09).

### 3.2. Predictors for Animal Reactions During the Milking Process

The results of the univariable analyses for predictors for the various reactions of interest observed on the farms are in Tables S6–S14. The results of the multivariable analyses are listed below.

#### 3.2.1. Proportion of Ewes That Displayed the Reactions ‘Kneeling’ Before Entry to the Milking Pen and ‘Kneeling’ Within the Milking Pen

**Figure 4:**
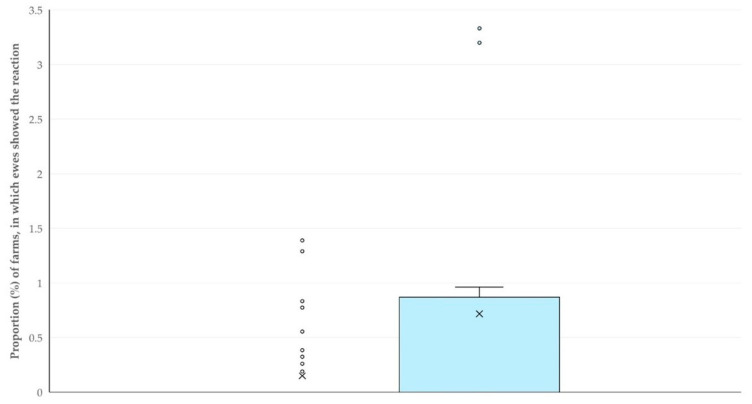
Box-and-whisker plot of the proportion of ewes that displayed the reaction ‘Kneeling’ before entry to the milking pen, in farms with (white dots) or without (light blue plot) a waiting area before the milking parlour.

The multivariable analysis revealed one predictor for the increased proportion of ewes that displayed the reaction ‘Kneeling’ before entry to the milking pen; specifically, the lack of a waiting area before the milking parlour (multivariable regression analysis, *p* = 0.026) (Table 6, Figure 4). Also, during the multivariable analysis, two predictors emerged for the increased proportion of ewes that displayed the reaction ‘Kneeling’ within the milking pen, specifically (i) the location of animals on the ground and milkers in the pit (multivariable regression analysis, *p* = 0.004) and (ii) no feed offered to drive ewes into the milking parlour (multivariable regression analysis, *p* = 0.007) (Table 7).

#### 3.2.2. Proportion of Ewes That Urinated or Defaecated

The multivariable analyses did not reveal any significant associations with the proportions of ewes that urinated or defaecated during the milking process. There was only a tendency for associating a lower pulse rate of the milking system with an increased proportion of ewes that urinated (multivariable regression analysis, *p* = 0.06) (Spearman’s rank correlation, *r_sp_* = −0.192, *p* = 0.17). Also, there was a tendency for associating a lower temperature in the milking parlour with an increased proportion of ewes that defaecated (multivariable regression analysis, *p* = 0.08) (Spearman’s rank correlation, *r_sp_* = −0.254, *p* = 0.07).

#### 3.2.3. Display of Vocalisation by Ewes

During the multivariable analysis, three predictors emerged for the display of vocalisation by ewes, specifically (i) the location of animals on ramp and milkers on ground (multivariable regression analysis, *p* = 0.008), (ii) increased total number of means used for driving ewes into the animal milking positions (multivariable regression analysis, *p* = 0.011) and (iii) shorter duration of the milking process (multivariable regression analysis, *p* = 0.013) (Table 8).

#### 3.2.4. Proportion of Ewes That Displayed Kick-like Reaction, That Attempted to Remove the Milking Cluster (Or Removed It) and That Showed Spot-Stepping

**Figure 5:**
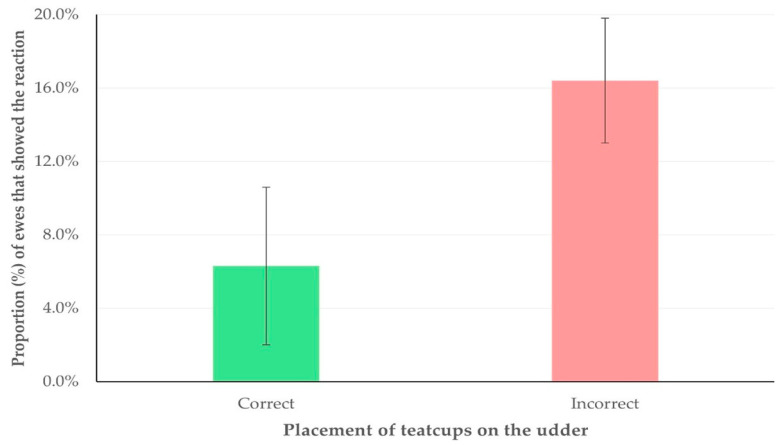
Proportion of ewes that displayed a kick-like reaction during the milking process, in accordance with correct (green) or incorrect (light red) placement of teatcups on the udder.

**Figure 6:**
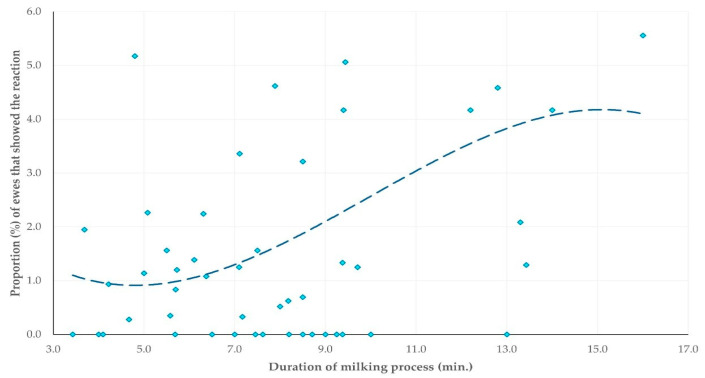
Scatter plot of the median duration of the milking process per milking row versus the proportion of ewes that attempted to remove the milking cluster (or removed it) during the milking process.

**Figure 7:**
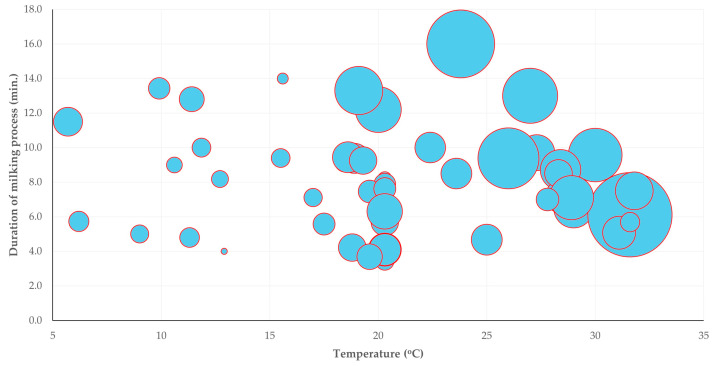
Bubble-plot of the temperature in the milking parlour and the median duration of the milking process versus the proportion of ewes that showed spot-stepping during the milking process (the diameter of bubbles reflects the proportion of ewes on a farm that showed a reaction).

The multivariable analysis revealed one predictor for the increased proportion of ewes that displayed kick-like reaction; specifically, the incorrect placement of teatcups (at least one) on the udder (i.e., lack of alignment and proper fitting onto the teats [28]) (multivariable regression analysis, *p* = 0.019) (Table 9, Figure 5), and another one predictor for the increased proportion of ewes that attempted to remove the milking cluster (or removed it); specifically, higher median duration of the milking process (multivariable regression analysis, *p* = 0.013) (Table 10, Figure 6). Moreover, during the multivariable analysis, three predictors emerged for the increased proportion of ewes that showed spot-stepping; specifically, (i) temperature in the milking parlour (multivariable regression analysis, *p* < 0.0001) and (ii) median duration of the milking process (multivariable regression analysis, *p* = 0.006) with a positive association, as well as (iii) early teatcup detachment (multivariable regression analysis, *p* = 0.014) with a negative association (Table 11, Figure 7).

**Figure 8:**
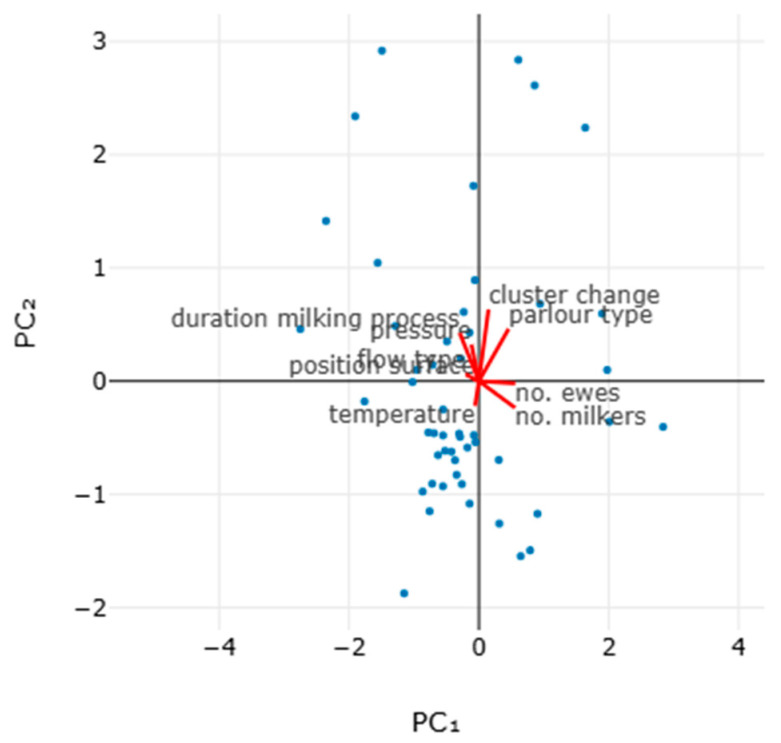
Bi-plot of results of principal component analysis for the proportion of ewes that attempted to remove the milking cluster (or removed it) during the milking process (vectors clockwise from top right: annual frequency of changing teatcups, type of milking parlour, number of ewes on the farm, number of working milkers, temperature in the milking parlour, material of surface of animal milking positions, type of flow line, system pressure, duration of milking process in each milking row) (standard scaling, with no rotation during preprocessing).

The principal component analysis for the proportion of ewes that attempted to remove the milking cluster indicated that the two principal components accounted for 44.0% of the variation (Table 12, Figure 8 and Figure S1).

**Figure 9:**
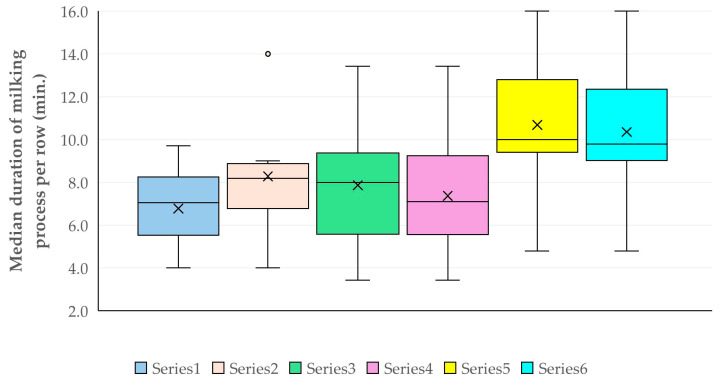
Box-and-whisker plot of the median duration of milking process per row in three cohorts of farms, defined in accordance with the proportion of ewes that displayed a kick-like reaction or spot-stepping and of those that attempted to remove the milking cluster (or removed it) (Series 1: Cohort with farms (cohort A) in which the proportion of ewes that displayed a kick-like reaction and of those that attempted to remove the milking cluster (or removed it) was below 4%; Series 2: Cohort with farms (cohort A) in which the proportion of ewes that displayed spot-stepping and of those that attempted to remove the milking cluster (or removed it) was below 4%; Series 3: Cohort with farms (cohort B) in which the proportion of ewes that displayed a kick-like reaction was over 4%, but the proportion of ewes that attempted to remove the milking cluster (or removed it) was below 4%; Series 4: Cohort with farms (cohort B) in which the proportion of ewes that displayed spot-stepping was over 4%, but the proportion of ewes that attempted to remove the milking cluster (or removed it) was below 4%; Series 5: Cohort with farms (cohort C) in which the proportion of ewes that displayed a kick-like reaction and of those that attempted to remove the milking cluster (or removed it) was over 4%; Series 6: Cohort with farms (cohort C) in which the proportion of ewes that displayed spot-stepping and of those that attempted to remove the milking cluster (or removed it) was over 4%).

The median duration of milking process per row was significantly longer in farms included in cohort C for (a) cohorts defined by displaying kick-like reaction and attempts to remove the milking cluster (multivariable regression analysis, *p* = 0.017) and (b) cohorts defined by displaying spot-stepping and attempts to remove the milking cluster (multivariable regression analysis, *p* = 0.015) (Table 13, Figure 9).

#### 3.2.5. Total Number of Distinct Reactions That Were Observed on Ewes on a Farm During the Milking Session

In the multivariable analysis, no independent variable emerged with significance as a predictor for the total number of distinct reactions that were observed in ewes. There was only a tendency for association with the total surface of animal-milking positions (multivariable regression analysis, *p* = 0.09).

### 3.3. Predictors for Somatic Cell Counts and Total Bacterial Counts

The results of the univariable analyses for predictors for somatic cell counts and total bacterial counts in the bulk-tank milk of the farms are in Tables S15 and S16. The results of the multivariable analyses are listed below.

#### 3.3.1. Somatic Cell Counts

**Figure 10:**
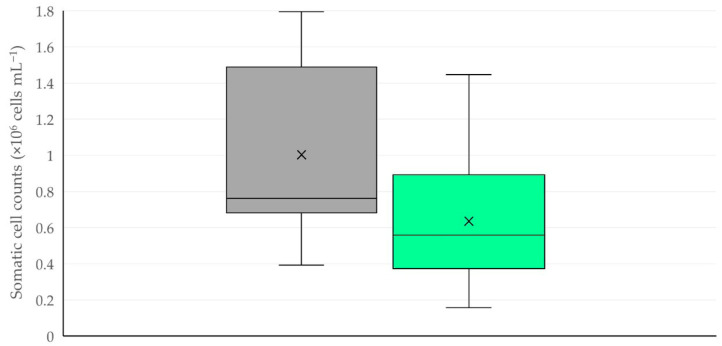
Box-and-whisker plot of the somatic cell counts in the bulk-tank milk in dairy sheep farms, where congestion of ewes before entry into the milking parlour was (grey) or was not (green) observed.

The multivariable analysis revealed one predictor for increased somatic cell counts in the bulk-tank milk, specifically the congestion of ewes before entry into the milking parlour (multivariable regression analysis, *p* = 0.047) (Table 14, Figure 10). In the analysis of clusters of farms, it emerged that the highest somatic cell counts were in farms with concurrent congestion of ewes before entry into the milking parlour and application of repeat milking: 1.749 × 10^6^ (IQR: 0.508 × 10^6^) (cells mL^−1^) (Kruskal–Wallis test, *p* = 0.041 between clusters) (Table 15).

#### 3.3.2. Total Bacterial Counts

**Figure 11:**
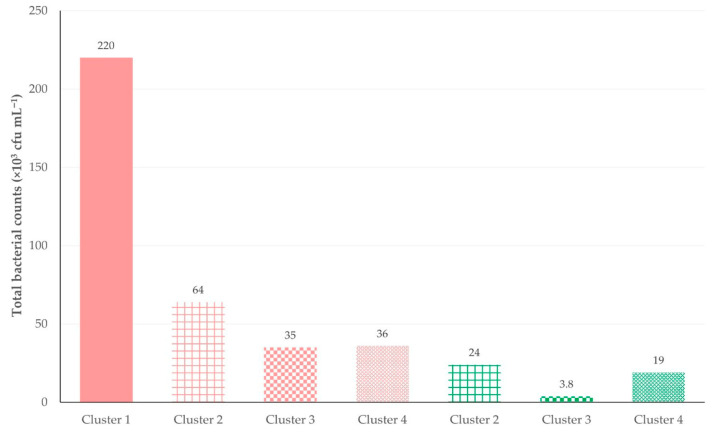
Clusters of farms in accordance with the variables included in the final model of multivariable analysis for total bacterial counts in the bulk-tank milk of dairy sheep farms (green/red bars: farms where post-milking teat disinfection was/was not performed; cluster 1: farms with a ratio <0.5; cluster 2: farms with a ratio = 0.5; cluster 3: farms with a ratio 0.5–1.0; cluster 4: farms with a ratio =1.0 for the number of milking units per animal-milking position in the parlour; numbers within the plot show median value of total bacterial counts (×10^3^ cfu ^2^ mL^−1^); cfu: colony-forming-units).

During the multivariable analysis, two predictors emerged for the increased total bacterial counts; specifically, (i) a smaller number of milking units per animal milking position in the milking parlour (multivariable regression analysis, *p* = 0.013) and (ii) omission of post-milking teat disinfection (multivariable regression analysis, *p* = 0.033) (Table 16). In the analysis of clusters of farms, it emerged that there was a reduction in total bacterial counts from cluster 4 to cluster 1 and, moreover, farms where teat disinfection was carried out had lower total bacterial counts independently of the cluster (Kruskal–Wallis test, *p* = 0.07) (Figure 11).

### 3.4. Recapitulation

In total, nine different predictors were identified for the displaying of the various reactions observed and recorded (Table 17). Of these, two predictors were related to the milking parlour and seven predictors were related to the actual milking process. Notably, the location of animal-milking positions in relation to work area of milkers and the increased median duration of the milking process per row were found to each be associated with two different reactions of ewes. Moreover, three different predictors were identified for the milk quality parameters (Table 17). Of these, one predictor was related to the milking parlour and two predictors were related to the actual milking process.

### 3.5. Correlations Between Animal Reactions and Characteristics of Bulk-Tank Milk

**Figure 12:**
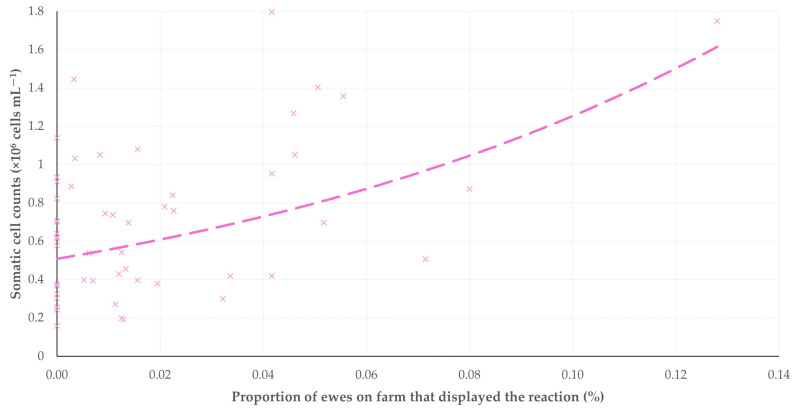
Scatter plot of the proportion of ewes that attempted to remove the milking cluster (or removed it) during the milking process versus the somatic cell counts in the bulk-tank milk on the same farm.

The findings of the correlation analyses of the reactions observed with the results of the milk testing revealed only one significant correlation. Specifically, there was a clear correlation between the proportion of ewes that attempted to remove the milking cluster (or removed it) and the somatic cell counts in the bulk-tank milk (Spearman’s rank correlation, *r_sp_* = 0.315, *p* = 0.023) (Figure 12). However, there was no correlation between the proportion of ewes that showed the above reaction and the total bacterial counts in the bulk-tank milk (Spearman’s rank correlation, *r_sp_* = −0.074, *p* = 0.60).

All other analyses did not yield statistically significant results (*p* ≥ 0.18 for correlations of proportions of ewes with somatic cell counts, *p* ≥ 0.20 for correlations of proportions of ewes with total bacterial counts) (Table S17).

## 4. Discussion

### 4.1. Preamble

Many recently published papers discuss stress factors in sheep, e.g., those by Tüfekci and Sejian [2], Tozlu Çelik et al. [29], Simeonov et al. [30], and Tada et al. [31]. Relevant publications deal with and discuss factors mostly related to transportation, weaning, shearing, handling, extreme temperatures and heat, feeding problems or diseases regarding the welfare of the animals. Nevertheless, other factors in the environment of the sheep may act as stressors for the animals; for example, recently, we identified the presence of canid predators near sheep farms [32] or co-grazing with wildlife ruminants [33] as potential stressors for sheep. Another little-studied potential stressor is the application of various reproductive techniques; among them, for example, intrauterine insemination [34,35].

All the above factors can have an adverse impact on outcomes related to the health and productivity of sheep, as they affect reproductive efficiency, reduced growth rate of lambs, and the milk production of ewes. Moreover, they may lead to an impairment of immune system, which would be followed by a higher susceptibility to infections.

The present work studied potential associations between factors related to the milking process in dairy sheep farms and reactions indicative of stress in the ewes being milked. This is a little studied topic, which is of particular importance in dairy sheep farms, as it relates to an important component of the management in these farms.

### 4.2. Comments on Potential Limitations

The inclusion of farms based on the willingness of farmers to participate (rather than through a randomised participation) might have introduced a bias in the study. Nevertheless, even under these circumstances when farms had been included on a convenience basis, we recorded farmers refusing to accept the visit after this had been arranged. Thus, one may postulate that, in cases of attempting to visit randomly selected farms of unknown farmers, they would be suspicious, unfriendly, and perhaps even hostile, which would result in a higher proportion of refusals.

As observations were performed during a single milk-collection season, potential differences in milking patterns and approaches, which occur with time and reflect various factors (for example, milk-selling price, the renewal of a flock through purchased animals and introduced replacement ewes), with possible influence on the reactions of ewes, might have been missed. The intention of the investigators was to extend the study with repeat visits during a second milking season, in that way collecting additional information. Unfortunately, though, this has not been possible due to the ongoing sheep pox outbreak in Greece, as the result of which very strict biosecurity measures have been enforced by veterinary authorities throughout the country. Consequently, all research visits to farms have been strictly prohibited.

Although the investigators remained aside from the main activity at the milking parlour during the study and only observed and recorded events occurring therein, one cannot rule out the possibility that their presence might have influenced, even to a minor degree, farmer actions or animal reactions. However, this is a standard limitation of all observational studies, in which the presence of observers is necessary for taking recordings.

Finally, only clinically evident reactions were recorded for evaluation of the animal welfare. Samples (e.g., blood, milk) from individual ewes, which would have provided additional information through the measurement of specific biomarkers (e.g., cortisol for assessment of possible stress, oxytocin for confirmation of the milk flow of ewes, milk for diagnosis of subclinical mastitis), were not collected. Indeed, the collection of samples from individual animals would have caused significant interference on the part of the investigators. This, in turn, would have resulted in a significant hinderance for the process and consequential delays; thus, it would have been impossible to calculate the various time intervals studied.

### 4.3. Reactions of Animals Recorded During the Milking Process and Potential Predictors

The results indicate that incorrect practices during machine-milking can be stress factors for dairy sheep and appear to cause some inconvenience or annoyance to the animals. This becomes evident as, during the milking process, ewes displayed a variety of reactions, which, in cattle and buffaloes, have been described as potential indicators of agitation and aggressiveness of the animals [11,15]. These include kick-like reactions and spot-stepping, which were the reactions mostly observed in the farms (>90.0%). The identification of the incorrect placement of teatcups (which has been previously described as a cause for inconvenience in animals [11]) as a predictor for kick-like reactions lends support to this argument. For this study, the correct placement of (both) teatcups on the udder referred to their alignment and proper fitting onto the teats [28]. The findings suggest that the ewes tried to remove the milking clusters during milking because they were in discomfort and possibly also in pain.

Sheep are sensitive to handling and to noise, two factors which abound during the milking process [2,36]. They can respond intensely to such events [37]. Therefore, reasonably, a longer duration of the milking process, i.e., a longer duration of such adverse stimuli, was found to be associated with a higher frequency of reactions by the animals. This was shown by the attempts of the ewes to remove the milking cluster from the udder: animals attempted to remove the teatcups as the inconvenience and their annoyance increased, and this consequently extended the duration of milking, which resulted in extended ‘handling’ and also extended exposure to the noises within the milking parlour. This is supported by the differences in the duration of milking time per row between the three cohorts of farms described in the study.

Moreover, the identification of the longer attachment of the teatcups on the udder as a predictor for increased spot-stepping activity, in which case the nuisance caused by the teatcups continued for a longer period, lends support to the above argument. This can lead to a possibly contradictory situation: early detachment of teacups during the milking process is unwanted, because it can adversely affect milking performance of dairy animals [38], but on the other hand, it contributes to a reduction in the reactions of the animals being milked.

In the majority of cases, the reactions recorded in the present study were associated with the legs of the animals. In addition to the above, another relevant reaction was the ‘kneeling’, which was recorded either before entry of the animals into the parlour or during the milking process. This was possibly the result of nervousness caused by the difficulty in entering into the parlour; indeed, Carcangiu et al. [20] reported higher cortisol levels in the blood of sheep that had to pass through a narrow entrance into the milking parlour. The reactions of the animals became more frequent in the presence of higher temperatures within the parlour, as observed with the identification of higher temperature as a predictor for increased spot-stepping. Although during high temperatures the movements, and activities in general, of sheep are generally reduced [39], one may postulate that the combination of adverse stimuli, i.e., teatcups on the udder for a prolonged period, coupled with a higher (micro)environmental temperature, aggravated the discomfort of the animals, which was displayed through the movement of their legs.

The increased frequency of reactions related to the legs and feet of the animals observed in the present study may possibly be the consequence of the primary annoyance caused during the milking process, an inconvenience exerted in the inguinal region. Thus, the animals reacted initially with their legs to reflect that (kick-like reactions, ‘spot-stepping’), whilst subsequently, if the adverse stimuli extended for a longer period, they attempted to alleviate the problem by trying to remove the teatcups (which might have resulted in early detachment of the teatcups).

In sheep, vocalisation has been previously used as an animal-based indicator of welfare-related issues [40,41]. In the present study, vocalisation was only occasionally observed (in less than 10% of farms) and was associated with a reduced duration of the milking process. In these same flocks, the farmers and milkers used a combination of means for driving ewes into the parlour (yells, twigs, whistles, etc.), aiming to quickly complete the milking process; this, on the one hand, contributed to a shorter duration of the process, but, on the other hand, created inconvenience to the animals, which reacted through the vocalisation. Frequent urination and defaecation have also been reported to indicate welfare issues [42], but in the current study, these reactions were observed only occasionally, and no particular predictor could be associated with them.

### 4.4. Somatic Cell Counts and Total Bacterial Counts and Potential Predictors

Previous studies on the somatic cell counts of milk produced on sheep farms focused on the effects of management factors applied in the farms [23,43,44]. In contrast, the current work focuses on the events that occur during the actual milking process, which is a particularly important aspect of dairy sheep farming. The findings provide evidence that animal stress derived from the incorrect practices during the process of machine-milking (in this case, the congestion of animals before entry to the milking parlour) can be associated with increased somatic cell counts in the bulk-tank milk produced in the farm. Hence, the findings indicate an association of the stress sustained by the animals during the milking procedures with elevated somatic cell counts in the milk produced during the milking. Τhis is further supported, first, by the results of the classification analysis, which indicated even higher somatic cell counts in cases of repeat milking (a practice applied in some farms in an attempt to maximise milk obtained from the animals), and, second, by the correlation of the somatic cell counts with the increased proportion of ewes attempting to remove the milking cluster.

In cattle, stress has been associated with high somatic cell counts in milk produced in a farm. For example, Hinks [45] reported that during the Second World War, plane bombings led to increasing cell content in the milk produced by cows located near those areas; in any case, it is hoped that there will be no opportunities to further study this factor! Later, Ling [46] reported that the worrying of milking cows by dogs might elicit changes in the characteristics of their milk. Wegner et al. [47] reported that stressing of cows by various factors present on the farm could lead to increased somatic cell counts in the absence of infection. Booth [48] introduced the broad term of ‘adverse environmental stimuli’ to provide a definition for these factors and indicated their potential consequences for increased somatic cell counts in the milk produced on the farm. These ‘environmental stimuli’ can include a variety of conditions; for example, adverse climatic conditions, among them increased heat and/or relative humidity [49,50,51].

In ewes, specific stressors found to lead to increased somatic cell counts in milk include the presence of canid predator animals (e.g., grey wolf) near farms; this has been associated with increased somatic cell counts in the bulk-tank milk without increased prevalence of subclinical mastitis on farms or high total bacterial counts in the bulk-tank milk [32]. Moreover, increased numbers of flies around sheep farms have also been associated with high somatic cell counts in the milk produced on these farms [52].

The correlation of the high somatic cell counts with a high proportion of ewes trying to remove the milking cluster supports the recognition of this reaction as indicative of stress in animals during milking. The stressing factors act through the hypothalamic–pituitary–adrenal axis and manifest through the increased numbers of blood leucocytes [47], subsequently leading to high somatic cell counts in milk [42,47].

Factors related to the milking process were also found to be associated with total bacterial counts, which is another indicator of the quality of milk produced in sheep farms. Teat disinfection contributes to a reduction in bacterial burdens on the farm [53], which explains its association with the lower total bacterial counts in the milk produced on the study farms. The total bacterial counts constitute the primary metric for the microbiological evaluation of the bulk-tank milk [54]. Moreover, a number of milking units equal to the that of milking positions indicates that milking units were used to milk a smaller number of animals; this contributes to the better functioning of the milking system [55], as well as to lower levels of dissemination of bacteria to animals during milking [56,57,58], which is reflected in the lower total bacterial counts in the bulk-tank milk of the farm.

## 5. Conclusions

The results confirmed that incorrect practices during the process of machine-milking process can act as a stress factor for dairy sheep. In light of the present findings, procedures should be carefully carried out by milkers and follow appropriate milking procedures, in order to minimise the nervousness and aggravation of ewes during milking.

Stress factors can adversely affect the quality of milk produced on the farm. This can be considered as analogous to the preslaughter stress and the adverse effects on the quality of meat further underline that the maintenance of animal welfare is linked to high product quality. This connection can further extend to consumer perceptions: the ethical treatment of dairy sheep is considered by consumers as an important facet of the overall food quality and sustainable production. The findings hold significant relevance for the welfare and production of all dairy animal species, and thus further research on this topic is valuable.

## Figures and Tables

**Table 1:** Description of time-events that were recorded during the monitoring of the milking session in dairy sheep farms.

Time-Event	Description
[1]	Ascent of the first ewe of each milking row onto the platform of the parlour, lined up for milking within the parlour
[2]	Placement of teatcups on the udder of the first ewe of each milking row (first ewe in the line-up within the parlour)
[3]	Removal of teatcups from the udder of the first ewe of each milking row (first ewe in the line-up within the parlour)
[4]	Removal of teatcups from the udder of the last ewe of each milking row (last ewe in milking process)
[5]	Release of ewes after completion of milking process in the milking row

**Table 2:** Ewe reactions that occurred and were recorded at an individual animal level during the milking session in dairy sheep farms.

Reaction	Description
‘Kneeling’ before entry to the milking pen	Touching at least one of the joints of the front or rear legs to the floor for at least 2 s, during walking towards the milking parlour
‘Kneeling’ within the milking pen	Touching at least one of the joints of the front or rear legs to the floor for at least 2 s, during the stay at the milking pen (before, during, or after the actual milking)
Urination	Urination during the stay at the milking pen (before, during, or after the actual milking)
Defaecation	Defaecation during the stay at the milking pen (before, during, or after the actual milking)
Vocalisation	Vocal reaction during the stay at the milking pen (before, during, or after the actual milking)
Kick-like reaction	Lifting a front or rear foot at least over the carpal/tarsal joint during the stay at the milking pen (before, during, or after the actual milking)
Attempting to remove the milking cluster	Touching the milking cluster with a rear foot and attempting to remove it (during the actual milking)
Displaying spot-stepping	Displaying walk-like movements of legs, imitating a ‘stationary walking’ (in the milking pen, before, during, or after the actual milking)

**Table 3:** Description of time intervals calculated for the milking process, based on recorded time-events, during the milking session in dairy sheep farms.

Start	End	Description
Within a milking row		
Time-event [1]	Time-event [2] ^1^	Duration of animal entrance into milking pens in a milking row (min.)
Time-event [2]	Time-event [3] ^1^	Duration of milking of first ewe in a milking row (min.)
Time-event [2]	Time-event [4] ^1^	Duration of milking process in a milking row (min.)
Time-event [4]	Time-event [5] ^1^	Duration of post-milking actions in a milking row (min.)
Within a milking session		
Time-event [1] of first milking row	Time-event [5] of last milking row ^1^	Duration of milking session on the farm (i.e., for all milking rows) (min.)

^1^ Description of the time-events used for the calculation of the time intervals is in Table 1.

**Table 4:** Time intervals calculated during the milking process in dairy sheep farms.

Time Intervals ^1^	Median Value
Period of preparation for milking process in each milking row	0.9 (0.7 ^2^) min.
Period of milking of first ewe in each milking row	3.0 (1.3) min.
Duration of milking process in each milking row	7.9 (3.7) min.
Duration of post-milking actions in each milking row	0.4 (0.3) min.
Duration of milking session on the farm	105.0 (51.8) min.

^1^ Descriptions of the time intervals and the time-events used for their calculation are in Table 1 and Table 3. ^2^ Interquartile range.

**Table 5:** Frequency of observation of ewes’ reactions during the milking process in dairy sheep farms.

Reaction	Farms (*n*), in Which Observed ^1^	Median Value Among Farms ^2^
‘Kneeling’ before entry to the milking pen	14 (26.9%)	0.0% (0.2% ^3^; 0.0–3.3% ^3^)
‘Kneeling’ within the milking pen	23 (46.2%)	0.0% (1.0%; 0.0–6.3%)
Urination	12 (23.1%)	0.0% (0.0%; 0.0–2.0%)
Defaecation	18 (34.6%)	0.0% (0.5%; 0.0–20.0%)
Vocalisation	5 (9.6%)	-- ^4^
Kick-like reaction	48 (92.3%)	7.1% (10.9%; 0.0–36.1%)
Attempting to remove the milking cluster (or removing it)	35 (67.3%)	1.1% (2.5%; 0.0–16.4%)
Displaying spot-stepping	51 (98.1%)	9.5% (11.3%; 0.0–95.0%)

^1^ Proportion among all farms in the study. ^2^ Median value of the proportion of ewes that displayed the reaction of interest during the milking process. ^3^ Interquartile range; min–max. ^4^ Not relevant.

**Table 6:** The results of the multivariable analysis for predictors associated with the proportion of ewes that displayed the reaction ‘Kneeling’ before entry to the milking pen.

Variables	Relative Risk (95% CI ^1^)	*p*
Availability of a waiting area		0.026
availability (*n* = 40, 0.0% (0.0%) ^2^)	reference	-
no availability (*n* = 12, 0.0% (0.7%))	1.006 (1.002–1.010)	0.011

^1^ Confidence interval. ^2^ Median (interquartile range).

**Table 7:** The results of the multivariable analysis for predictors associated with the proportion of ewes that displayed the reaction ‘Kneeling’ within the milking pen.

Variables	Relative Risk (95% CI ^1^)	*p*
Location of animal-milking positions in relation to work area of milkers		0.004
animals on ramp and milkers on ground (*n* = 23, 0.0% (0.7%) ^2^)	reference	-
animals on ground and milkers in a pit (*n* = 29, 0.0% (1.7%))	1.008 (1.001–1.014)	0.025
Offering of feed as a means for driving ewes into the animal-milking positions		0.007
offering of feed (*n* = 50, 0.0% (0.9%) ^2^)	reference	-
no offering of feed (*n* = 2, 1.1% (1.2%))	1.011 (1.001–1.021)	0.043

^1^ Confidence interval. ^2^ Median (interquartile range).

**Table 8:** The results of the multivariable analysis for predictors associated with the display of vocalisation by ewes.

Variables	Odds Ratio/Relative Risk (95% CI ^1^)	*p*
Location of animal-milking positions in relation to work area of milkers		0.008
animals on ramp and milkers on ground (*n* = 23, 100.0%)	10.551 (0.552–201.568)	0.12
animals on ground and milkers in a pit (*n* = 29, 51.1%)	reference	-
Total no. of means used for driving ewes into the animal-milking positions		0.011
per unit (means) increase	1.134 (1.010–1.274)	0.034
Duration of milking session on the farm		0.013
per unit (min.) decrease	1.002 (0.999–1.004)	0.06

^1^ Confidence interval.

**Table 9:** The results of the multivariable analysis for predictors associated with the proportion of ewes that displayed kick-like reaction during the milking process.

Variables	Relative Risk (95% CI ^1^)	*p*
Correct placement of teatcups on the udder		0.019
Yes (*n* = 45, 6.3% (8.5%) ^2^)	reference	-
No (*n* = 7, 16.4% (6.8%))	1.097 (1.032–1.166)	0.004

^1^ Confidence interval. ^2^ Median (interquartile range).

**Table 10:** The results of the multivariable analysis for predictors associated with the proportion of ewes that attempted to remove the milking cluster (or removed it) during the milking process.

Variables	Relative Risk (95% CI ^1^)	*p*
Median duration of milking process per milking row		0.013
per unit (min.) increase	1.003 (1.001–1.007)	0.007

^1^ Confidence interval.

**Table 11:** The results of the multivariable analysis for predictors associated with the proportion of ewes that showed spot-stepping during the milking process.

Variables	Relative Risk (95% CI ^1^)	*p*
Temperature in the milking parlour		<0.0001
per unit (°C) increase	1.013 (1.006–1.019)	0.0001
Median duration of milking process per milking row		0.006
per unit (min.) increase	1.016 (1.000–1.032)	0.05
Early teatcup detachment		0.014
Yes (*n* = 48, 9.0% (9.7%) ^2^)	reference	
No (*n* = 4, 29.0% (40.2%) ^2^)	1.307 (1.111–1.537)	0.002

^1^ Confidence interval. ^2^ Median (interquartile range).

**Table 12:** Eigenvalues for principal component analysis for the proportion of ewes that attempted to remove the milking cluster (or removed it) during the milking process.

Parameter	PC1	PC2	PC3	PC4	PC5	PC6	PC7	PC8	PC9
Eigenvalue	2.53	1.43	1.37	1.04	0.85	0.76	0.60	0.26	0.15
% of variance	28.1	15.9	15.3	11.5	9.4	8.4	6.7	2.9	1.7
Cumulative variance (%)	28.1	44.0	59.3	70.8	80.3	88.7	95.4	98.3	100

**Table 13:** Time intervals (median (interquartile range)) regarding the milking process in three cohorts of farms, defined in accordance with the proportion of ewes that displayed a kick-like reaction or spot-stepping and of those that attempted to remove the milking cluster (or removed it).

Cohorts Defined Based on Displaying a Kick-like Reaction and Attempts to Remove the Milking Cluster				
Time intervals ^1^	A ^2^ (*n* = 19)	B (*n* = 23)	C (*n* = 10)	*p*
Period of preparation for milking process in each milking row	0.9 (0.8) ^3^	1.0 (0.7)	0.8 (1.9)	0.80
Period of milking of first ewe in each milking row	2.8 (1.4)	3.0 (1.1)	3.5 (2.5)	0.32
Duration of milking process in each milking row	7.1 (2.6)	8.0 (3.7)	10.0 (3.1)	0.015
Duration of post-milking actions in each milking row	0.6 (0.8)	0.4 (0.1)	0.3 (0.1)	0.048
**Cohorts Defined Based on** **Displaying Spot-Stepping and Attempts to Remove the Milking Cluster**				
Time intervals ^1^	A ^2^ (*n* = 8)	B (*n* = 34)	C (*n* = 10)	*p*
Period of preparation for milking process in each milking row	1.3 (0.9) ^3^	0.9 (0.8)	0.8 (1.9)	0.24
Period of milking of first ewe in each milking row	2.7 (1.0)	3.0 (1.2)	3.5 (2.5)	0.19
Duration of milking process in each milking row	8.2 (1.0)	7.1 (3.5)	9.8 (2.6)	0.015
Duration of post-milking actions in each milking row	0.4 (0.3)	0.4 (0.6)	0.3 (0.1)	0.19

^1^ Descriptions of the time intervals and the time-events used for their calculation are in Table 1 and Table 3. ^2^ Cohort A included farms in which the proportion of ewes that displayed a kick-like reaction or spot-stepping and of those that attempted to remove the milking cluster (or removed it) was below 4%; cohort B included farms in which proportion of ewes that displayed a kick-like reaction or spot-stepping was over 4%, but the proportion of ewes that attempted to remove the milking cluster (or removed it) was below 4%; cohort C included farms in which proportion of ewes that displayed a kick-like reaction or spot-stepping and of those that attempted to remove the milking cluster (or removed it) was over 4%. ^3^ Values expressed in min.

**Table 14:** The results of the multivariable analysis for predictors associated with somatic cell counts in the bulk-tank milk in dairy sheep farms.

Variables	Relative Risk (95% CI ^1^)	*p*
Congestion of ewes before entry into the milking parlour		0.047
Yes (*n* = 10, 0.763 × 10^6^ (0.635 × 10^6^) cells mL^−1 2^)	2.054 (1.160–3.634)	0.015
No (*n* = 42, 0.559 × 10^6^ (0.506 × 10^6^) cells mL^−1^)	reference	-

^1^ Standard error. ^2^ Median (interquartile range).

**Table 15:** Clusters of farms in accordance with the variables included in the final model of multivariable analysis for somatic cell counts in the bulk-tank milk of dairy sheep farms.

Cluster	*n*	Variables			Somatic Cell Counts (Cells mL^−1^) ^2^
		Facilities for Milk Yield Measurement	Congestion of Ewes Before Entry into mp ^1^	Repeat Milking of Ewes	
I	34	No	No	No	0.595 × 10^6^ (IQR: 0.489 × 10^6^)
II	3	Yes	No	No	0.417 × 10^6^ (IQR: 0.131 × 10^6^)
III	7	No	Yes	No	0.707 × 10^6^ (IQR: 0.281 × 10^6^)
IV	5	No	No	Yes	0.934 × 10^6^ (IQR: 0.542 × 10^6^)
V	3	No	Yes	Yes	1.749 × 10^6^ (IQR: 0.508 × 10^6^)

^1^ mp = milking parlour. ^2^ Median (interquartile range).

**Table 16:** The results of the multivariable analysis for predictors associated with total bacterial counts in the bulk-tank milk in dairy sheep farms.

Variables	Relative Risk (95% CI ^1^)	*p*
Number of animal milking positions for a milking unit		0.013
per unit (animal position) increase	1.072 (0.975–1.178)	0.15
Post-milking teat disinfection		0.033
Yes (*n* = 10, 18 × 10^3^ (49 × 10^3^) cfu ^2^ mL^−1 3^)	reference	-
No (*n* = 42, 62 × 10^3^ (118 × 10^3^) cfu mL^−1^)	1.500 (0.982–2.291)	0.06

^1^ Standard error. ^2^ cfu = colony-forming units. ^3^ Median (interquartile range).

**Table 17:** Predictors identified with an association to animal reactions during the milking process and with the characteristics of milk produced in dairy sheep farms.

Predictors	Animal Reactions
Lack of availability of a waiting area before the milking parlour	‘Kneeling’ before entry to the milking pen
Location of animal milking positions in relation to work area of milkers	‘Kneeling’ within the milking pen
	Display of vocalisation
No offering of feed as a means for driving ewes into the animal milking positions	‘Kneeling’ within the milking pen
Total no. of means used for driving ewes into the animal milking positions	Display of vocalisation
Increased temperature in the milking parlour	Spot-stepping
Incorrect placement of teatcups on the udder	Kick-like reaction
Avoidance of early teatcup detachment	Spot-stepping
Increased duration of the milking process per row	Attempt to remove the milking cluster
	Spot-stepping
Duration of milking session on the farm	Display of vocalisation
**Predictors**	**Characteristics of Milk Quality**
Number of animal-milking positions for a milking unit	Total bacterial counts
Congestion of ewes before entry into the milking parlour	Somatic cell counts
Post-milking teat disinfection	Total bacterial counts

## Data Availability

Most of the data presented in this study are contained within the text and in the Supplementary Materials. The remaining data are available on request from the corresponding author. The data are not publicly available as they form part of the PhD thesis of the first author, which has not yet been examined, approved, and uploaded in the official depository of PhD theses from Greek Universities.

## Supplementary Materials

The following supporting information can be downloaded at: https://www.mdpi.com/article/10.3390/ani15213078/s1, Table S1: Details of information recorded at sheep farms during visits to monitor the milking process in dairy sheep farms; Table S2: List of independent variables for the evaluation of predictors for reactions of ewes during the milking process in dairy sheep farms; Table S3: Details of multivariable models employed for the evaluation of potential associations with ewe reactions that occurred during the milking session in dairy sheep farms; Table S4: Details of multivariable models employed for the evaluation of potential associations with parameters related to quality of the bulk-tank milk in dairy sheep farms; Table S5: Details of findings regarding (a) general information about the farm and the farmer, (b) information about the milking parlour, (c) information about the milking process in 52 dairy sheep farms in Greece; Table S6: Results of univariable analysis for potential associations between the independent variables and the outcome % of ewes that displayed ‘Kneeling’ before entry to the milking pen; Table S7: Results of univariable analysis for potential associations between the independent variables and the outcome % of ewes that displayed ‘Kneeling’ within the milking pen; Table S8: Results of univariable analysis for potential associations between the independent variables and the outcome % of ewes that urinated; Table S9: Results of univariable analysis for potential associations between the independent variables and the outcome % of ewes that defaecated; Table S10: Results of univariable analysis for potential associations between the independent variables and the outcome display of vocalisation by at least one ewe; Table S11: Results of univariable analysis for potential associations between the independent variables and the outcome % of ewes that displayed kick-like reaction; Table S12: Results of univariable analysis for potential associations between the independent variables and the outcome % of ewes that attempted to remove the milking cluster (or removed it); Table S13: Results of univariable analysis for potential associations between the independent variables and the outcome % of ewes that showed spot-stepping; Table S14: Results of univariable analysis for potential associations between the independent variables and the outcome total number of distinct reactions observed on ewes on a farm during the milking session; Figure S1: Scree-plot for principal component analysis for the proportion of ewes that attempted to remove the milking cluster (or removed it) during the milking process; Table S15: Results of univariable analysis for potential associations between the independent variables and the somatic cell counts in the bulk-tank milk; Table S16: Results of univariable analysis for potential associations between the independent variables and the total bacterial counts in the bulk-tank milk; Table S17: Results of correlation analyses for potential associations between the various reactions observed in ewes during the milking process and the characteristics of bulk-tank milk produced on the farms.

## References

[1] Kilgour R. (1975). The open-field test as an assessment of the temperament of dairy cows. Anim. Behav..

[2] Tüfekci H., Sejian V. (2023). Stress factors and their effects on productivity in sheep. Animals.

[3] Blokhuis H.J., Hopster H., Geverink N.A., Korte S.M., Van Reenen C.G. (1998). Studies of stress in farm animals. Comp. Haematol. Int..

[4] Yardimci M., Sahin E.H., Cetingul I.S., Bayram I., Aslan R., Sengor E. (2013). Stress responses to comparative handling procedures in sheep. Animal.

[5] Unsal G., Johnson K.F., Stergiadis S., Bennett R., Barker Z.E. (2025). A systematic review and meta-analysis of physical environmental enrichment to improve animal welfare-related outcomes in indoor cattle. Anim. Welf..

[6] Lawrence A.B., Terlouw E.M.C., Illius A.W. (1991). Individual differences in behavioural responses of pigs exposed to non-social and social challenges. Appl. Anim. Behav. Sci..

[7] Dwyer C.M. (2008). The Welfare of Sheep.

[8] Grandin T., Shivley C. (2015). How farm animals react and perceive stressful situations such as handling, restraint, and transport. Animals.

[9] Van Reenen C.G., Van Der Werf J.T.N., Bruckmaier R.M., Hopster H., Engel B., Noordhuizen J.P.T.M., Blokhuis H.J. (2002). Individual differences in behavioral and physiological responsiveness of primiparous dairy cows to machine milking. J. Dairy Sci..

[10] Saltalamacchia F., Tripaldi C., Castellano A., Napolitano F., Musto M., De Rosa G. (2007). Human and animal behaviour in dairy buffalo at milking. Anim. Welf..

[11] Cavallina R., Roncoroni C., Campagna M.C., Minero M., Canali E. (2008). Buffalo behavioural response to machine milking in early lactation. It. J. Anim. Sci..

[12] Andrioli M., Grajales-Cedeño J.K., Sant’Anna A.C., Negrão J.A., Paranhos da Costa M.J.R. (2024). Milking reactivity in primiparous saanen goats during early lactation: Effects on milk yield, milk quality and plasma cortisol concentration. Animals.

[13] Kézér F.L., Kovács L., Tőzsér J. (2015). Step behaviour and autonomic nervous system activity in multiparous dairy cows during milking in a herringbone milking system. Animal.

[14] Hemsworth P.H., Coleman G.J., Barnett J.L., Borg S. (2000). Relationships between human-animal interactions and productivity of commercial dairy cows. J. Anim. Sci..

[15] Munksgaard L., DePassillé A.M., Rushen J., Herskin M.S., Kristensen A.M. (2001). Dairy cows’ fear of people: Social learning, milk yield and behaviour at milking. Appl. Anim. Behav. Sci..

[16] De Rosa G., Napolitano F., Grasso F., Pacelli C., Bordi A. (2005). On the development of a monitoring scheme of buffalo welfare at farm level. It. J. Anim. Sci..

[17] Faid-Allah E., Mourad R.S., Saddick E.I., Eldahshan E. (2025). Managerial factors affecting milking-abilities of Holstein cattle under intensive production system in Egypt. Trop. Anim. Health Prod..

[18] Rushen J., Munksgaard L., Marnet P.G., DePassillé A.M. (2001). Human contact and the effects of acute stress on cows at milking. Appl. Anim. Behav. Sci..

[19] Pacheco H.A., Hernandez R.O., Chen S.Y., Neave H.W., Pempek J.A., Brito L.F. (2025). Phenotyping strategies and genetic background of dairy cattle behavior in intensive production systems—From trait definition to genomic selection. J. Dairy Sci..

[20] Carcangiu V., Arfuso F., Luridiana S., Giannetto C., Rizzo M., Bini P.P., Piccione G. (2018). Relationship between different livestock managements and stress response in dairy ewes. Arch. Anim. Breed..

[21] European Food Safety Authority (2014). Scientific opinion on the welfare risks related to the farming of sheep for wool, meat and milk production. EFSA J..

[22] Lianou D.T., Chatziprodromidou I.P., Vasileiou N.G.C., Michael C.K., Mavrogianni V.S., Politis A.P., Kordalis N.G., Billinis C., Giannakopoulos A., Papadopoulos E. (2020). A detailed questionnaire for the evaluation of health management in dairy sheep and goats. Animals.

[23] Lianou D.T., Michael C.K., Vasileiou N.G.C., Petinaki E., Cripps P.J., Tsilipounidaki K., Katsafadou A.I., Politis A.P., Kordalis N.G., Ioannidi K.S. (2021). Extensive countrywide field investigation of somatic cell counts and total bacterial counts in bulk-tank raw milk in sheep flocks in Greece. Foods.

[24] Laird D.T., Gambrel-Lenarz S.A., Scher F.M., Graham T.E., Reddy R., Wehr H.M., Frank J.F. (2004). Microbiological Count Methods. Standard Methods for the Examination of Dairy Products.

[25] Dohoo I., Martin W., Stryhn H. (2014). Veterinary Epidemiologic Research.

[26] Wiggans G.R., Shook G.E. (1987). A lactation measure of somatic cell count. J. Dairy Sci..

[27] Franzoi M., Manuelian C.L., Penasa M., De Marchi M. (2020). Effects of somatic cell score on milk yield and mid-infrared predicted composition and technological traits of Brown Swiss, Holstein Friesian, and Simmental cattle breeds. J. Dairy Sci..

[28] Blowey R., Edmondson P. (2010). Mastitis Control in Dairy Herds.

[29] Tozlu Çelik H., Aslan F.A., Us Altay D., Kahveci M.E., Konanc K., Noyan T., Ayhan S. (2021). Effects of transport and altitude on hormones and oxidative stress parameters in sheep. PLoS ONE.

[30] Simeonov M.S., Stoycheva I., Harmon D.L. (2022). Environmental temperature influences diet selection and growth in early-weaned lambs. Ir. J. Appl. Anim. Sci..

[31] Tada O., Tshabuse P.M., Mamakoko M.S., Mashamaite P.K. (2025). Evaluation of stress hormones on reproductive functions of sheep and goats: A systematic review. Front. Anim. Sci..

[32] Katsarou E.I., Reid N., Lianou D.T., Fthenakis G.C. (2024). Stress related to wild canid predators near dairy sheep farms associated with increased somatic cell counts in bulk-tank milk. Sci. Rep..

[33] Katsarou E.I. (2025). A Study of Ecosystemic Interactions in Small Ruminant Farms Within the Context of One Health and Precision Medicine: Environmental Variables, Climatic Influence, Health Problems and Diseases, Quality of Milk, Interactions with Domesticated and Wild Animals, Development of Prediction Models. Ph.D. Thesis.

[34] Khalid M., Haresign W., Bradley D.G. (1998). Heart rate responses and plasma cortisol concentrations in ewes: Comparison between cervical and laparoscopic intrauterine insemination and their associated handling procedures. Anim. Sci..

[35] El Amiri B., Rahim A. (2024). Exploring endogenous and exogenous factors for successful artificial insemination in sheep: A global overview. Vet. Sci..

[36] Arfuso F., Fazio F., Chikhi L., Aymond G., Piccione G., Giannetto C. (2022). Acute stress response of sheep to shearing procedures: Dynamic change of cortisol concentration and protein electrophoretic pattern. Animals.

[37] Hemsworth P.H., Rice M., Borg S., Edwards L.E., Ponnampalam E.N., Coleman G.J. (2019). Relationships between handling, behaviour and stress in lambs at abattoirs. Animal.

[38] Lüdi I., Bruckmaier R.M. (2022). The teat cup detachment level affects milking performance in an automatic milking system with teat cleaning and milking in the same teat cup. J. Dairy Res..

[39] Leu S.T., Quiring K., Leggett K.E.A., Griffith S.C. (2021). Consistent behavioural responses to heatwaves provide body condition benefits in rangeland sheep. Appl. Anim. Behav. Sci..

[40] Richmond S.E., Wemelsfelder F., De Heredia I.B., Ruiz R., Canali E., Dwyer C.M. (2017). Evaluation of animal-based indicators to be used in a welfare assessment protocol for sheep. Front. Vet. Sci..

[41] Zufferey R., Minnig A., Thomann B., Zwygart S., Keil N., Schüpbach G., Miserez R., Zanolari P., Stucki D. (2021). Animal-based indicators for on-farm welfare assessment in sheep. Animals.

[42] Caroprese M., Casamassima D., Pier Giacomo Rassu S., Napolitano F., Sevi A. (2009). Monitoring the on-farm welfare of sheep and goats. J. Anim. Sci..

[43] Albenzio M., Figliola L., Caroprese M., Marino R., Sevi A., Santillo A. (2019). Somatic cell count in sheep milk. Small Rumin. Res..

[44] Gonzalo C., Juarez M.T., Garcia-Jimeno M.C., De La Fuente L.F. (2019). Bulk tank somatic cell count and total bacterial count are affected by target practices and milking machine features in dairy sheep flocks in Castilla y Leon region, Spain. Small Rumin. Res..

[45] Hinks E. (1941). The effect of air raids on the composition of milk. Anal. Lond..

[46] Ling E.R. (1956). A Textbook of Dairy Chemistry.

[47] Wegner T.N., Schuh J.D., Nelson F.E., Stott G.H. (1974). Effect of stress on blood leucocyte and milk somatic cell counts in dairy cows. J. Dairy Sci..

[48] Booth J.M. (1975). Cell counting in milk. Vet. A.

[49] Smith D.L., Smith T., Rude B.J., Ward S.H. (2013). Comparison of the effects of heat stress on milk and component yields and somatic cell score in Holstein and Jersey cows. J. Dairy Sci..

[50] Guinn J.M., Nolan D.T., Krawczel P.D., Petersson-Wolfe C.S., Pighetti G.M., Stone A.E., Ward S.H., Bewley J.M., Costa J.H.C. (2019). Comparing dairy farm milk yield and components, somatic cell score, and reproductive performance among United States regions using summer to winter ratios. J. Dairy Sci..

[51] Negri R., dos Santos Daltro D., Araújo Cobuci J. (2021). Heat stress effects on somatic cell score of Holstein cattle in tropical environment. Livest. Sci..

[52] Arsenopoulos K.V., Sioutas G., Triantafillou E., Gelasakis A.I., Papadopoulos E. (2021). Will fly repellency using deltamethrin reduce intramammary infections, stress and fatigue indicators of dairy ewes under intensive management?. Pathogens.

[53] Ózsvári L., Ivanyos D. (2022). The use of teat disinfectants and milking machine cleaning products in commercial Holstein-Friesian farms. Front. Vet. Sci..

[54] Twomey L., Furey A., O’Brien B., Beresford T., Gleeson D. (2025). Minimizing bacterial counts in bulk tank milk: A review with a focus on chlorine-free cleaning. Dairy.

[55] Caria M., Todde G., Pazzona A. (2020). Influence of the milking units on the pulsation curve in dairy sheep milking. Animals.

[56] Magee C., Sagi R., Scott N.R., Gates R.S. (1984). Bacterial transport within and among various teatcup and cluster assemblies during milking. J. Dairy Sci..

[57] Romero G., Peris C., Fthenakis G.C., Diaz J.R. (2020). Effects of machine milking on udder health in dairy ewes. Small Rumin. Res..

[58] Michael C.K., Lianou D.T., Tsilipounidaki K., Gougoulis D.A., Giannoulis T., Vasileiou N.G.C., Mavrogianni V.S., Petinaki E., Fthenakis G.C. (2023). Recovery of staphylococci from teatcups in milking parlours in goat herds in Greece: Prevalence, identification, biofilm formation, patterns of antibiotic susceptibility, predictors for isolation. Antibiotics.

