# Supplementary material for: Machine-Milking Practices, Animal Welfare-Related Reactions and Quality of Milk Produced in Dairy Sheep Farms
Source: Animals (Basel). 2025 Oct 23;15(21):3078. doi: 10.3390/ani15213078 (PMC12609417; doi:10.3390/ani15213078)
Supplement: Supplementary file 1 [file animals-15-03078-s001.zip › animals-3880693-supplementary.pdf]

Machine-Milking Practices, Animal Welfare-Related Reactions and Quality of Milk Produced in Dairy Sheep Farms

Dimitra V. Liagka, George C. Fthenakis, Stella N. Kalonaki, Konstantina S. Dimoveli, Daphne T. Lianou, Vasia S. Mavrogianni, Charalambia K. Michael, Mariangela Caropresе, Vassiliki Spyrou, Natalia G. C. Vasileiou

Table S1. Details of information recorded at sheep farms during visits to monitor the milking process in dairy sheep farms.

No.	Information
	General
01	Farm reference (no.)
02	National identification of the farm (no.)
03	Name of the farmer (name)
04	Address (description)
05	Date of the visit (date)
06	Start date of the most recent lambing season (date)
	Animals
07	Number of ewes on the farm (no.)
08	Number of ewes to be milked during the visit (no.)
09	Breed of ewes (description)
	Farmer
10	Age of farmer (years)
11	Previous animal farming experience (yes / no)
12	Length of previous animal farming experience (years)
13	General education (yes / no)
14	General education (description)
	Milking parlour
15	Year of initial establishment (year)
16	Availability of a waiting area before the milking parlour (yes / no)
17	Availability of ventilators (yes / no)
18	Type of milking parlour (circular / parallel / linear / other)
19	Location of animal milking positions in relation to work area of milkers (animals on ramp and milkers on ground / animals on ground and milkers into a pit)
20	Number of animal milking positions in the parlour (no.)
21	Dimensions of animal milking positions (length × width in m)
22	Material of surface of animal milking positions (cement / tile / soil / other)
23	Smoothness of surface of animal milking positions (yes / no)
24	Number of available milking units (no.)
25	Teatcup material (description)
26	System pulsation rate (p. min^{-1})
27	System pressure (kPa)
28	Type of flow line (low / high)
29	Availability of facilities for milk yield measurement (yes / no)
30	Type of facilities for milk yield measurement (individual / group)
31	Provision of concentrate feed during milking (yes / no)
32	Proportion of concentrate feed provided during milking of total daily allowance of concentrate feed (%)
33	Availability of automatic removal of clusters (yes / no)
34	'Fast-exit' facility of ewes from the milking position (yes / no)
35	Criteria for changing teatcups (description)

36	Annual frequency of changing teatcups (no. of occasions)
	Milking process
37	Ewe congestion before entry into the milking parlour (yes / no)
38	Means for driving ewes into the animal milking positions (feed / whistle / flexible twig / yells)
39	People participating in driving ewes into the animal milking positions (no.)
40	Number of working milkers (no.)
41	Gender of working milkers (male / female)
42	Apparel of milkers (description)
43	Use of gloves by milkers (yes / no)
44	Temperature in the milking parlour (^{o}C)
45	Relative humidity in the milking parlour (%)
46	Radio playing within the milking parlour (yes / no)
47	Application of pre-milking stripping of ewes (yes / no)
48	Correct placement of teatcups on the udder (yes / no)
49	Yells from milkers (yes / no)
50	Early teatcup detachment (yes / no)
51	End-of-milking stripping of ewes (yes / no)
52	'Two-phase' milking of ewes (yes / no)
53	Post-milking teat disinfection (yes / no)
54	Technique applied for post-milking teat disinfection (dipping / spraying)
55	Delayed animal exit (yes / no)

Table S2. List of independent variables for the evaluation of predictors for reactions of ewes during the milking process in dairy sheep farms.

Independent variables
Variables related to general information about the farm and the farmer (*n* = 6)
Number of ewes on the farm (no.)
Breed of ewes (description)
Milking session (morning / evening)
Age of farmer (years)
Length of previous animal farming experience (years)
Level of general education of farmer (primary, secondary, tertiary, professional)
Variables related to information about the milking parlour (*n* =15)
Year of initial establishment (year)
Availability of a waiting area before the milking parlour (yes / no)
Availability of ventilators (yes / no)
Type of milking parlour (circular / parallel / linear / other)
Location of animal milking positions in relation to work area of milkers (animals on ramp and milkers on ground / animals on ground and milkers into a pit)
Surface of animal milking positions (m^2)
Material of surface of animal milking positions (cement / tile / soil / other)
Smoothness of surface of animal milking positions (yes / no)
Number of milking units per animal milking position in the parlour (ratio: 'Number of available milking units / Number of animal milking positions in the parlour')
Teatcup material (description)
System pulsation rate (p. min^{-1})
System pressure (kPa)
Type of flow line (low / high)
Availability of facilities for milk yield measurement (yes / no)
Availability of automatic removal of clusters (yes / no)
Frequency of replacement of milking clusters (no. of occasions annually)
Variables related to information about the milking process (*n* = 26)
Ewe congestion before entry into the milking parlour (yes / no)
Means for driving ewes into the animal milking positions (feed, yes / no)
Means for driving ewes into the animal milking positions (whistle, yes / no)
Means for driving ewes into the animal milking positions (flexible twig, yes/ no)
Means for driving ewes into the animal milking positions (yells, yes / no)
Means for driving ewes into the animal milking positions (total no. of means used for driving ewes, no.)
People participating in driving ewes into the animal milking positions (no.)
Number of working milkers (no.)
Use of gloves by milkers (yes / no)
Provision of concentrate feed during milking (yes / no)
Temperature in the milking parlour (°C) (median value of three recordings)
Relative humidity in the milking parlour (%) (median value of three reconrdings)
Radio playing within the milking parlour (yes / no)
Application of pre-milking stripping of ewes (yes / no)
Correct placement of teatcups on the udder (yes / no)
Yells from milkers (yes / no)
Early teatcup detachment (yes / no)
End-of-milking stripping of ewes (yes / no)
Repeat milking of ewes (yes / no)
Post-milking teat disinfection (yes / no)
Delayed animal exit (yes / no)
Period of preparation for milking process in each milking row
Period of milking for first ewe in each milking row
Duration of milking process in each milking row
Duration of post-milking actions in each milking row
Duration of milking session in the farm

Table S3.

Details of multivariable models (n = 8) employed for the evaluation of potential associations with ewe reactions that occurred during the milking session in dairy sheep farms.

Outcome	Variables		
	assessed in uni-variable analyses (n)	offered to the multi-variable models (n)	required in the final models
'Kneeling' before entry to the milking parlour	48	14	(a) Year of initial establishment, (b) Availability of a waiting area before the milking parlour, (c) System pulsation rate
'Kneeling' within the milking pen	48	8	(a) Means for driving ewes into the animal milking positions (feed), (b) Location of animal milking positions in relation to work area of milkers
Urination	48	13	(a) System pulsation rate, (b) Delayed animal exit
Defaecation	48	18	(a) Breed of ewes, (b) System pulsation rate, (c) Number of mil-kers active, (d) Temperature in the milking parlour
Display of vocalisation	48	15	(a) Location of animal milking positions in relation to work area of milkers, (b) Means for driving ewes into the animal milking positions (total), (c) Relative humidity in the milking parlour, (d) Delayed animal exit, (e) Duration of milking session in the farm
Displaying kick-like reaction	48	21	(a) Length of farmer's previous animal farming experience, (b) Means for driving ewes into the animal milking positions (yells), (c) Correct placement of teatcups on the udder, (d) Repeat milking of ewes, (e) Delayed animal exit
Attempting to remove the milking cluster (or removing it)	48	9	(a) System pressure, (b) Temperature in the milking parlour, (c) Duration of milking process in each milking row
Displaying spot stepping	48	19	(a) Type of milking parlour, (b) System pulsation rate, (c) Means for driving ewes into the animal milking positions (feed), (d) People participating in driving ewes into the animal milking positions, (e) Temperature in the milking parlour, (f) Early teatcup detachment, (g) Duration of milking process in each milking row
Total number of distinct reactions in ewes during the milking session	48	8	(a) Surface of animal milking positions

Table S4.

Details of multivariable models (n = 2) employed for the evaluation of potential associations with parameters related to quality of the bulk-tank milk in dairy sheep farms.

Outcome	Variables		
	assessed in uni-variable analyses (*n*)	offered to the multi-variable models (*n*)	required in the final models
Somatic cell counts	48	6	(a) Availability of facilities for milk yield measurement, (b) Ewe congestion before entry into the milking parlour, (c) Repeat milking of ewes
Total bacterial counts	48	12	(a) Number of milking units per ani-mal milking position in the parlour, (b) Post-milking teat disinfection

Table S5.

Details of findings regarding (a) general information about the farm and the farmer, (b) information about the milking parlour, (c) information about the milking process in 52 dairy sheep farms in Greece.

Number of ewes on the farm (no.)			
400 (220)			
Breed of ewes (description)			
Chios<br>*n* = 3	Lacaune<br>*n* = 7	Local<br>*n* = 2	Cross-breeds<br>*n* = 40
Milking session			
Morning<br>*n* = 17	Evening<br>*n* = 35		
Age of farmer (years)			
43 (15)			
Length of previous animal farming experience (years)			
17 (10.5)			
Level of general education of farmer			
Secondary<br>*n* = 31	Tertiary<br>*n* = 15	Professional<br>*n* = 6	
Year of initial establishment (year)			
2012 (10.5 years)			
Availability of a waiting area before the milking parlour			
Yes<br>*n* = 40	No<br>*n* = 12		
Availability of ventilators			
Yes<br>*n* = 37	No<br>*n* = 15		
Type of milking parlour			
Circular<br>*n* = 1	Parallel<br>*n* = 12	Linear<br>*n* = 39	
Location of animal milking positions in relation to work area of milkers			
Animals on ramp and milkers on ground<br>*n* = 22	Animals on ground and milkers into a pit<br>*n* = 29		
Surface of animal milking positions (m^2)			
3745 (909)			
Material of surface of animal milking positions			
Cement<br>*n* = 29	Tile<br>*n* = 11	Steel<br>*n* = 3	Wood<br>*n* = 9
Smoothness of surface of animal milking positions (yes / no)			
Yes<br>*n* = 8	No<br>*n* = 44		
Number of milking units per animal milking position in the parlour (ratio)			
0.8 (0.5)			
Teatcup material			
Silicone<br>*n* = 51	Nitrile rubber<br>*n* = 1		
System pulsation rate (p. min^{-1})			
150 (20)			
System pressure (kPa)			
38.5 (1)			
Type of flow line			
Low line<br>*n* = 43	High line<br>*n* = 6	Mobile unit<br>*n* = 3	
Availability of facilities for milk yield measurement			
Yes<br>*n* = 3	No<br>*n* = 49		

Availability of automatic removal of clusters	
Yes	No
n = 0	*n* = 52
Frequency of replacement of milking clusters (no. of occasions annually)	
1.0 (0.5)	
Ewe congestion before entry into the milking parlour	
Yes	No
n = 10	*n* = 42
Means for driving ewes into the animal milking positions (feed)	
Yes	No
n = 46	*n* = 6
Means for driving ewes into the animal milking positions (whistle)	
Yes	No
n = 12	*n* = 40
Means for driving ewes into the animal milking positions (flexible twig)	
Yes	No
n = 13	*n* = 39
Means for driving ewes into the animal milking positions (yells)	
Yes	No
n = 11	*n* = 41
Means for driving ewes into the animal milking positions (total no. of means used for driving ewes)	
1 (1)	
People participating in driving ewes into the animal milking positions (no.)	
1 (0)	
Number of working milkers (no.)	
2 (1)	
Use of gloves by milkers	
Yes	No
n = 35	*n* = 17
Provision of concentrate feed during milking	
Yes	No
n = 50	*n* = 2
Temperature in the milking parlour (°C) (median value of three recordings)	
20,5 (10)	
Relative humidity in the milking parlour (%) (median value of three reconrdings)	
59 (24)	
Radio playing within the milking parlour	
Yes	No
n = 25	*n* = 27
Application of pre-milking stripping of ewes	
Yes	No
n = 0	*n* = 52
Correct placement of teatcups on the udder	
Yes	No
n = 45	*n* = 7
Yells from milkers	
Yes	No
n = 8	*n* = 44
Early teatcup detachment	
Yes	No
n = 48	*n* = 4
End-of-milking stripping of ewes	
Yes	No
n = 45	*n* = 7
Repeat milking of ewes	
Yes	No
n = 8	*n* = 44

Post-milking teat disinfection	
Yes<br>*n* = 10	No<br>*n* = 42
**Delayed animal exit**	
Yes<br>*n* = 14	No<br>*n* = 38
**Period of preparation for milking process in each milking row (min.)**	
0.9 (0.7)	
**Period of milking for first ewe in each milking row (min.)**	
3.0 (1.3)	
**Duration of milking process in each milking row (min.)**	
7.9 (3.7)	
**Duration of post-milking actions in each milking row (min.)**	
0.4 (0.3)	
**Duration of milking session in the farm (min.)**	
105.0 (51.8)	

Table S6.

Results of univariable analysis for potential associations between the independent variables and the outcome % of ewes that displayed 'Kneeling' before entry to the milking pen.

Independent variables (*n* = 48)	r_{sp}	*p*
Number of ewes on the farm (no.)	0.140	0.32
Breed of ewes (description)	0.070	0.63
Milking session (morning / evening)	0.107	0.45
Age of farmer (years)	0.111	0.43
Length of previous animal farming experience (years)	0.155	0.27
Level of general education of farmer (primary, secondary, tertiary, professional)	−0.229	0.10
Year of initial establishment (year)	0.252	0.07
Availability of a waiting area before the milking parlour (yes / no)	−0.224	0.11
Availability of ventilators (yes / no)	0.043	0.76
Type of milking parlour (circular / parallel / linear / other)	−0.140	0.32
Location of animal milking positions in relation to work area of milkers (animals on ramp and milkers on ground / animals on ground and milkers into a pit)	0.165	0.24
Surface of animal milking positions (m^2)	0.243	0.09
Material of surface of animal milking positions (cement / tile / soil / other)	0.008	0.96
Smoothness of surface of animal milking positions (yes / no)	−0.111	0.43
Number of milking units per animal milking position in the parlour (ratio)	0.218	0.12
Teatcup material (description)	−0.083	0.56
System pulsation rate (p. min^{-1})	0.268	0.05
System pressure (kPa)	−0.091	0.52
Type of flow line (low / high)	−0.044	0.76
Availability of facilities for milk yield measurement (yes / no)	0.039	0.78
Availability of automatic removal of clusters (yes / no)	n/r	n/r
Frequency of replacement of milking clusters (no. of occasions annually)	−0.016	0.91
Ewe congestion before entry into the milking parlour (yes / no)	−0.006	0.97
Means for driving ewes into the animal milking positions (feed, yes / no)	−0.010	0.94
Means for driving ewes into the animal milking positions (whistle, yes / no)	0.051	0.72
Means for driving ewes into the animal milking positions (flexible twig, yes/ no)	0.296	0.033
Means for driving ewes into the animal milking positions (yells, yes / no)	0.026	0.85
Means for driving ewes into the animal milking positions (total no. of means used for driving ewes, no.)	0.217	0.12
People participating in driving ewes into the animal milking positions (no.)	−0.160	0.26
Number of working milkers (no.)	−0.378	0.79
Use of gloves by milkers (yes / no)	−0.308	0.026
Provision of concentrate feed during milking (yes / no)	0.119	0.40
Temperature in the milking parlour (°C) (median value of three recordings)	0.069	0.63
Relative humidity in the milking parlour (%) (median value of three reconrdings)	−0.109	0.44
Radio playing within the milking parlour (yes / no)	−0.110	0.44
Application of pre-milking stripping of ewes (yes / no)	n/r	n/r
Correct placement of teatcups on the udder (yes / no)	0.084	0.55
Yells from milkers (yes / no)	−0.018	0.89
Early teatcup detachment (yes / no)	0.172	0.22
End-of-milking stripping of ewes (yes / no)	−0.209	0.14
Repeat milking of ewes (yes / no)	−0.018	0.89
Post-milking teat disinfection (yes / no)	0.040	0.78
Delayed animal exit (yes / no)	0.283	0.042
Period of preparation for milking process in each milking row	−0.120	0.40
Period of milking for first ewe in each milking row	0.231	0.10
Duration of milking process in each milking row	−0.078	0.58
Duration of post-milking actions in each milking row	0.312	0.024
Duration of milking session in the farm	0.189	0.18

Table S7.

Results of univariable analysis for potential associations between the independent variables and the outcome % of ewes that displayed 'Kneeling' within the milking pen'

Independent variables (n = 48)	r_{sp}	p
Number of ewes on the farm (no.)	−0.154	0.27
Breed of ewes (description)	0.070	0.63
Milking session (morning / evening)	−0.105	0.46
Age of farmer (years)	−0.021	0.88
Length of previous animal farming experience (years)	−0.091	0.52
Level of general education of farmer (primary, secondary, tertiary, professional)	−0.122	0.39
Year of initial establishment (year)	0.056	0.69
Availability of a waiting area before the milking parlour (yes / no)	−0.144	0.31
Availability of ventilators (yes / no)	−0.114	0.42
Type of milking parlour (circular / parallel / linear / other)	−0.248	0.08
Location of animal milking positions in relation to work area of milkers (animals on ramp and milkers on ground / animals on ground and milkers into a pit)	0.228	0.10
Surface of animal milking positions (m^2)	0.129	0.36
Material of surface of animal milking positions (cement / tile / soil / other)	0.141	0.32
Smoothness of surface of animal milking positions (yes / no)	0.012	0.93
Number of milking units per animal milking position in the parlour (ratio)	0.082	0.56
Teatcup material (description)	0.077	0.59
System pulsation rate (p. min^{-1})	0.102	0.47
System pressure (kPa)	−0.006	0.97
Type of flow line (low / high)	−0.111	0.43
Availability of facilities for milk yield measurement (yes / no)	0.142	0.32
Availability of automatic removal of clusters (yes / no)	n/r	n/r
Frequency of replacement of milking clusters (no. of occasions annually)	−0.027	0.85
Ewe congestion before entry into the milking parlour (yes / no)	−0.086	0.50
Means for driving ewes into the animal milking positions (feed, yes / no)	−0.201	0.14
Means for driving ewes into the animal milking positions (whistle, yes / no)	0.243	0.08
Means for driving ewes into the animal milking positions (flexible twig, yes/ no)	0.007	0.96
Means for driving ewes into the animal milking positions (yells, yes / no)	0.105	0.45
Means for driving ewes into the animal milking positions (total no. of means used for driving ewes, no.)	0.133	0.35
People participating in driving ewes into the animal milking positions (no.)	−0.237	0.09
Number of working milkers (no.)	−0.129	0.85
Use of gloves by milkers (yes / no)	−0.410	0.003
Provision of concentrate feed during milking (yes / no)	0.169	0.23
Temperature in the milking parlour (°C) (median value of three recordings)	0.007	0.96
Relative humidity in the milking parlour (%) (median value of three reconrdings)	−0.047	0.74
Radio playing within the milking parlour (yes / no)	0.001	0.99
Application of pre-milking stripping of ewes (yes / no)	n/r	n/r
Correct placement of teatcups on the udder (yes / no)	−0.014	0.92
Yells from milkers (yes / no)	0.039	0.78
Early teatcup detachment (yes / no)	0.156	0.27
End-of-milking stripping of ewes (yes / no)	0.120	0.40
Repeat milking of ewes (yes / no)	0.127	0.37
Post-milking teat disinfection (yes / no)	0.061	0.67
Delayed animal exit (yes / no)	−0.222	0.88
Period of preparation for milking process in each milking row	−0.127	0.38
Period of milking for first ewe in each milking row	0.267	0.06
Duration of milking process in each milking row	0.123	0.39
Duration of post-milking actions in each milking row	0.059	0.68
Duration of milking session in the farm	0.223	0.11

Table S8.

Results of univariable analysis for potential associations between the independent variables and the outcome % of ewes that urinated.

Independent variables (*n* = 48)	r_{sp}	*p*
Number of ewes on the farm (no.)	−0.165	0.24
Breed of ewes (description)	−0.049	0.73
Milking session (morning / evening)	−0.252	0.07
Age of farmer (years)	−0.029	0.84
Length of previous animal farming experience (years)	−0.029	0.84
Level of general education of farmer (primary, secondary, tertiary, professional)	0.159	0.26
Year of initial establishment (year)	0.033	0.82
Availability of a waiting area before the milking parlour (yes / no)	0.078	0.58
Availability of ventilators (yes / no)	0.029	0.83
Type of milking parlour (circular / parallel / linear / other)	−0.0001	0.99
Location of animal milking positions in relation to work area of milkers (animals on ramp and milkers on ground / animals on ground and milkers into a pit)	−0.115	0.42
Surface of animal milking positions (m^2)	0.404	0.003
Material of surface of animal milking positions (cement / tile / soil / other)	0.123	0.39
Smoothness of surface of animal milking positions (yes / no)	0.038	0.79
Number of milking units per animal milking position in the parlour (ratio)	−0.040	0.78
Teatcup material (description)	0.284	0.041
System pulsation rate (p. min^{-1})	−0.192	0.17
System pressure (kPa)	−0.128	0.37
Type of flow line (low / high)	−0.141	0.32
Availability of facilities for milk yield measurement (yes / no)	−0.1334	0.35
Availability of automatic removal of clusters (yes / no)	n/r	n/r
Frequency of replacement of milking clusters (no. of occasions annually)	0.018	0.90
Ewe congestion before entry into the milking parlour (yes / no)	0.029	0.84
Means for driving ewes into the animal milking positions (feed, yes / no)	0.084	0.55
Means for driving ewes into the animal milking positions (whistle, yes / no)	−0.010	0.94
Means for driving ewes into the animal milking positions (flexible twig, yes/ no)	0.120	0.40
Means for driving ewes into the animal milking positions (yells, yes / no)	0.096	0.50
Means for driving ewes into the animal milking positions (total no. of means used for driving ewes, no.)	0.165	0.24
People participating in driving ewes into the animal milking positions (no.)	−0.247	0.08
Number of working milkers (no.)	−0.026	0.85
Use of gloves by milkers (yes / no)	−0.366	0.007
Provision of concentrate feed during milking (yes / no)	0.108	0.45
Temperature in the milking parlour (°C) (median value of three recordings)	−0.282	0.043
Relative humidity in the milking parlour (%) (median value of three reconrdings)	0.187	0.18
Radio playing within the milking parlour (yes / no)	0.307	0.027
Application of pre-milking stripping of ewes (yes / no)	n/r	n/r
Correct placement of teatcups on the udder (yes / no)	−0.061	0.67
Yells from milkers (yes / no)	0.190	0.18
Early teatcup detachment (yes / no)	−0.036	0.80
End-of-milking stripping of ewes (yes / no)	−0.046	0.75
Repeat milking of ewes (yes / no)	0.108	0.45
Post-milking teat disinfection (yes / no)	0.103	0.47
Delayed animal exit (yes / no)	−0.329	0.017
Period of preparation for milking process in each milking row	0.117	0.41
Period of milking for first ewe in each milking row	0.031	0.81
Duration of milking process in each milking row	0.331	0.017
Duration of post-milking actions in each milking row	−0.212	0.13
Duration of milking session in the farm	0.046	0.75

Table S9.

Results of univariable analysis for potential associations between the independent variables and the outcome % of ewes that defaecated.

Independent variables (*n* = 48)	r_{sp}	*p*
Number of ewes on the farm (no.)	−0.212	0.13
Breed of ewes (description)	0.216	0.12
Milking session (morning / evening)	−0.329	0.017
Age of farmer (years)	0.380	0.79
Length of previous animal farming experience (years)	0.014	0.93
Level of general education of farmer (primary, secondary, tertiary, professional)	0.206	0.14
Year of initial establishment (year)	−0.324	0.019
Availability of a waiting area before the milking parlour (yes / no)	−0.023	0.87
Availability of ventilators (yes / no)	−0.040	0.78
Type of milking parlour (circular / parallel / linear / other)	0.130	0.36
Location of animal milking positions in relation to work area of milkers (animals on ramp and milkers on ground / animals on ground and milkers into a pit)	−0.135	0.34
Surface of animal milking positions (m^2)	−0.185	0.19
Material of surface of animal milking positions (cement / tile / soil / other)	−0.016	0.91
Smoothness of surface of animal milking positions (yes / no)	0.027	0.85
Number of milking units per animal milking position in the parlour (ratio)	−0.096	0.50
Teatcup material (description)	0.269	0.05
System pulsation rate (p. min^{-1})	−0.186	0.19
System pressure (kPa)	0.152	0.28
Type of flow line (low / high)	−0.223	0.11
Availability of facilities for milk yield measurement (yes / no)	0.197	0.16
Availability of automatic removal of clusters (yes / no)	n/r	n/r
Frequency of replacement of milking clusters (no. of occasions annually)	0.184	0.19
Ewe congestion before entry into the milking parlour (yes / no)	−0.153	0.28
Means for driving ewes into the animal milking positions (feed, yes / no)	0.106	0.45
Means for driving ewes into the animal milking positions (whistle, yes / no)	−0.113	0.43
Means for driving ewes into the animal milking positions (flexible twig, yes/ no)	−0.049	0.73
Means for driving ewes into the animal milking positions (yells, yes / no)	0.091	0.52
Means for driving ewes into the animal milking positions (total no. of means used for driving ewes, no.)	−0.026	0.85
People participating in driving ewes into the animal milking positions (no.)	−0.012	0.93
Number of working milkers (no.)	−0.185	0.19
Use of gloves by milkers (yes / no)	0.101	0.48
Provision of concentrate feed during milking (yes / no)	0.141	0.32
Temperature in the milking parlour (°C) (median value of three recordings)	−0.254	0.07
Relative humidity in the milking parlour (%) (median value of three reconrdings)	0.076	0.59
Radio playing within the milking parlour (yes / no)	0.092	0.52
Application of pre-milking stripping of ewes (yes / no)	n/r	n/r
Correct placement of teatcups on the udder (yes / no)	−0.091	0.52
Yells from milkers (yes / no)	0.105	0.46
Early teatcup detachment (yes / no)	0.204	0.15
End-of-milking stripping of ewes (yes / no)	−0.060	0.67
Repeat milking of ewes (yes / no)	−0.209	0.14
Post-milking teat disinfection (yes / no)	0.088	0.53
Delayed animal exit (yes / no)	−0.202	0.15
Period of preparation for milking process in each milking row	0.342	0.013
Period of milking for first ewe in each milking row	0.207	0.14
Duration of milking process in each milking row	0.149	0.29
Duration of post-milking actions in each milking row	−0.177	0.21
Duration of milking session in the farm	0.135	0.34

Table S10.

Results of univariable analysis for potential associations between the independent variables and the outcome display of vocalisation by at least one ewe.

Independent variables (n = 48)	r_{sp}	p
Number of ewes on the farm (no.)	0.109	0.44
Breed of ewes (description)	−0.162	0.25
Milking session (morning / evening)	−0.190	0.18
Age of farmer (years)	0.072	0.61
Length of previous animal farming experience (years)	0.112	0.43
Level of general education of farmer (primary, secondary, tertiary, professional)	0.119	0.40
Year of initial establishment (year)	−0.190	0.17
Availability of a waiting area before the milking parlour (yes / no)	0.179	0.21
Availability of ventilators (yes / no)	−0.368	0.007
Type of milking parlour (circular / parallel / linear / other)	0.107	0.45
Location of animal milking positions in relation to work area of milkers (animals on ramp and milkers on ground / animals on ground and milkers into a pit)	−0.290	0.037
Surface of animal milking positions (m^2)	0.219	0.12
Material of surface of animal milking positions (cement / tile / soil / other)	−0.555	0.70
Smoothness of surface of animal milking positions (yes / no)	−0.139	0.33
Number of milking units per animal milking position in the parlour (ratio)	0.088	0.54
Teatcup material (description)	−0.046	0.75
System pulsation rate (p. min^{-1})	−0.132	0.35
System pressure (kPa)	0.077	0.59
Type of flow line (low / high)	−0.149	0.29
Availability of facilities for milk yield measurement (yes / no)	−0.081	0.57
Availability of automatic removal of clusters (yes / no)	n/r	n/r
Frequency of replacement of milking clusters (no. of occasions annually)	0.188	0.19
Ewe congestion before entry into the milking parlour (yes / no)	0.006	0.97
Means for driving ewes into the animal milking positions (feed, yes / no)	−0.086	0.54
Means for driving ewes into the animal milking positions (whistle, yes / no)	−0.024	0.87
Means for driving ewes into the animal milking positions (flexible twig, yes/ no)	0.264	0.06
Means for driving ewes into the animal milking positions (yells, yes / no)	0.310	0.025
Means for driving ewes into the animal milking positions (total no. of means used for driving ewes, no.)	0.272	0.05
People participating in driving ewes into the animal milking positions (no.)	−0.149	0.29
Number of working milkers (no.)	0.024	0.87
Use of gloves by milkers (yes / no)	0.088	0.53
Provision of concentrate feed during milking (yes / no)	−0.274	0.049
Temperature in the milking parlour (^{o}C) (median value of three recordings)	−0.070	0.62
Relative humidity in the milking parlour (%) (median value of three reconrdings)	0.242	0.08
Radio playing within the milking parlour (yes / no)	0.078	0.58
Application of pre-milking stripping of ewes (yes / no)	n/r	n/r
Correct placement of teatcups on the udder (yes / no)	0.129	0.36
Yells from milkers (yes / no)	0.223	0.11
Early teatcup detachment (yes / no)	0.094	0.51
End-of-milking stripping of ewes (yes / no)	−0.062	0.66
Repeat milking of ewes (yes / no)	−0.139	0.33
Post-milking teat disinfection (yes / no)	0.006	0.97
Delayed animal exit (yes / no)	−0.198	0.16
Period of preparation for milking process in each milking row	0.030	0.83
Period of milking for first ewe in each milking row	0.087	0.83
Duration of milking process in each milking row	−0.059	0.68
Duration of post-milking actions in each milking row	−0.083	0.56
Duration of milking session in the farm	−0.309	0.026

Table S11.

Results of univariable analysis for potential associations between the independent variables and the outcome % of ewes that displayed kick-like reaction.

Independent variables (n = 48)	r_{sp}	p
Number of ewes on the farm (no.)	−0.499	0.0002
Breed of ewes (description)	0.255	0.07
Milking session (morning / evening)	−0.348	0.011
Age of farmer (years)	−0.185	0.19
Length of previous animal farming experience (years)	−0.291	0.036
Level of general education of farmer (primary, secondary, tertiary, professional)	0.241	0.09
Year of initial establishment (year)	−0.169	0.24
Availability of a waiting area before the milking parlour (yes / no)	−0.114	0.42
Availability of ventilators (yes / no)	0.071	0.62
Type of milking parlour (circular / parallel / linear / other)	−0.333	0.016
Location of animal milking positions in relation to work area of milkers (animals on ramp and milkers on ground / animals on ground and milkers into a pit)	0.053	0.71
Surface of animal milking positions (m^2)	−0.038	0.79
Material of surface of animal milking positions (cement / tile / soil / other)	0.092	0.52
Smoothness of surface of animal milking positions (yes / no)	−0.028	0.84
Number of milking units per animal milking position in the parlour (ratio)	−0.360	0.009
Teatcup material (description)	0.014	0.92
System pulsation rate (p. min^{-1})	0.009	0.95
System pressure (kPa)	0.100	0.48
Type of flow line (low / high)	0.234	0.09
Availability of facilities for milk yield measurement (yes / no)	0.066	0.64
Availability of automatic removal of clusters (yes / no)	n/r	n/r
Frequency of replacement of milking clusters (no. of occasions annually)	0.119	0.40
Ewe congestion before entry into the milking parlour (yes / no)	−0.257	0.07
Means for driving ewes into the animal milking positions (feed, yes / no)	−0.008	0.96
Means for driving ewes into the animal milking positions (whistle, yes / no)	0.046	0.75
Means for driving ewes into the animal milking positions (flexible twig, yes/ no)	−0.334	0.016
Means for driving ewes into the animal milking positions (yells, yes / no)	0.242	0.08
Means for driving ewes into the animal milking positions (total no. of means used for driving ewes, no.)	−0.041	0.77
People participating in driving ewes into the animal milking positions (no.)	−0.160	0.26
Number of working milkers (no.)	−0.436	0.001
Use of gloves by milkers (yes / no)	0.031	0.83
Provision of concentrate feed during milking (yes / no)	0.007	0.96
Temperature in the milking parlour (^{o}C) (median value of three recordings)	−0.259	0.06
Relative humidity in the milking parlour (%) (median value of three reconrdings)	0.084	0.55
Radio playing within the milking parlour (yes / no)	0.026	0.85
Application of pre-milking stripping of ewes (yes / no)	n/r	n/r
Correct placement of teatcups on the udder (yes / no)	−0.347	0.012
Yells from milkers (yes / no)	−0.092	0.52
Early teatcup detachment (yes / no)	0.141	0.32
End-of-milking stripping of ewes (yes / no)	0.058	0.68
Repeat milking of ewes (yes / no)	0.195	0.17
Post-milking teat disinfection (yes / no)	0.332	0.016
Delayed animal exit (yes / no)	−0.508	0.0001
Period of preparation for milking process in each milking row	0.124	0.38
Period of milking for first ewe in each milking row	0.108	0.45
Duration of milking process in each milking row	0.266	0.06
Duration of post-milking actions in each milking row	−0.390	0.005
Duration of milking session in the farm	−0.185	0.19

Table S12.

Results of univariable analysis for potential associations between the independent variables and the outcome % of ewes that attempted to remove the milking cluster (or removed it).

Independent variables (*n* = 48)	r_{sp}	*p*
Number of ewes on the farm (no.)	−0.216	0.12
Breed of ewes (description)	0.098	0.49
Milking session (morning / evening)	−0.053	0.71
Age of farmer (years)	0.035	0.81
Length of previous animal farming experience (years)	−0.015	0.92
Level of general education of farmer (primary, secondary, tertiary, professional)	0.011	0.94
Year of initial establishment (year)	0.000	0.99
Availability of a waiting area before the milking parlour (yes / no)	0.017	0.9
Availability of ventilators (yes / no)	0.078	0.58
Type of milking parlour (circular / parallel / linear / other)	−0.209	0.14
Location of animal milking positions in relation to work area of milkers (animals on ramp and milkers on ground / animals on ground and milkers into a pit)	−0.028	0.85
Surface of animal milking positions (m^2)	0.227	0.11
Material of surface of animal milking positions (cement / tile / soil / other)	−0.016	0.91
Smoothness of surface of animal milking positions (yes / no)	0.094	0.51
Number of milking units per animal milking position in the parlour (ratio)	0.055	0.7
Teatcup material (description)	0.052	0.71
System pulsation rate (p. min^{-1})	0.090	0.49
System pressure (kPa)	0.215	0.13
Type of flow line (low / high)	−0.201	0.15
Availability of facilities for milk yield measurement (yes / no)	0.076	0.59
Availability of automatic removal of clusters (yes / no)	n/r	n/r
Frequency of replacement of milking clusters (no. of occasions annually)	0.211	0.14
Ewe congestion before entry into the milking parlour (yes / no)	−0.007	0.96
Means for driving ewes into the animal milking positions (feed, yes / no)	−0.131	0.35
Means for driving ewes into the animal milking positions (whistle, yes / no)	0.003	0.98
Means for driving ewes into the animal milking positions (flexible twig, yes/ no)	0.095	0.50
Means for driving ewes into the animal milking positions (yells, yes / no)	0.152	0.28
Means for driving ewes into the animal milking positions (total no. of means used for driving ewes, no.)	0.075	0.59
People participating in driving ewes into the animal milking positions (no.)	−0.145	0.31
Number of working milkers (no.)	−0.254	0.07
Use of gloves by milkers (yes / no)	−0.053	0.71
Provision of concentrate feed during milking (yes / no)	−0.156	0.27
Temperature in the milking parlour (^{o}C) (median value of three recordings)	−0.204	0.15
Relative humidity in the milking parlour (%) (median value of three reconrdings)	0.041	0.77
Radio playing within the milking parlour (yes / no)	−0.136	0.34
Application of pre-milking stripping of ewes (yes / no)	n/r	n/r
Correct placement of teatcups on the udder (yes / no)	−0.120	0.40
Yells from milkers (yes / no)	0.112	0.43
Early teatcup detachment (yes / no)	0.062	0.68
End-of-milking stripping of ewes (yes / no)	−0.105	0.46
Repeat milking of ewes (yes / no)	0.143	0.31
Post-milking teat disinfection (yes / no)	0.238	0.09
Delayed animal exit (yes / no)	−0.068	0.63
Period of preparation for milking process in each milking row	0.122	0.39
Period of milking for first ewe in each milking row	0.076	0.59
Duration of milking process in each milking row	0.280	0.044
Duration of post-milking actions in each milking row	0.038	0.79
Duration of milking session in the farm	−0.056	0.69

Table S13.

Results of univariable analysis for potential associations between the independent variables and the outcome % of ewes that showed spot stepping.

Independent variables (*n* = 48)	r_{sp}	*p*
Number of ewes on the farm (no.)	−0.233	0.10
Breed of ewes (description)	0.121	0.39
Milking session (morning / evening)	0.496	0.0002
Age of farmer (years)	0.275	0.048
Length of previous animal farming experience (years)	0.124	0.38
Level of general education of farmer (primary, secondary, tertiary, professional)	−0.454	< 0.0001
Year of initial establishment (year)	−0.111	0.43
Availability of a waiting area before the milking parlour (yes / no)	−0.097	0.49
Availability of ventilators (yes / no)	0.205	0.14
Type of milking parlour (circular / parallel / linear / other)	−0.207	0.14
Location of animal milking positions in relation to work area of milkers (animals on ramp and milkers on ground / animals on ground and milkers into a pit)	0.166	0.24
Surface of animal milking positions (m^2)	0.177	0.21
Material of surface of animal milking positions (cement / tile / soil / other)	0.044	0.76
Smoothness of surface of animal milking positions (yes / no)	−0.179	0.21
Number of milking units per animal milking position in the parlour (ratio)	−0.011	0.93
Teatcup material (description)	0.051	0.72
System pulsation rate (p. min^{-1})	0.435	0.001
System pressure (kPa)	0.123	0.39
Type of flow line (low / high)	0.047	0.74
Availability of facilities for milk yield measurement (yes / no)	−0.300	0.031
Availability of automatic removal of clusters (yes / no)	n/r	n/r
Frequency of replacement of milking clusters (no. of occasions annually)	0.044	0.76
Ewe congestion before entry into the milking parlour (yes / no)	0	0.99
Means for driving ewes into the animal milking positions (feed, yes / no)	−0.233	0.09
Means for driving ewes into the animal milking positions (whistle, yes / no)	0.398	0.003
Means for driving ewes into the animal milking positions (flexible twig, yes/ no)	0.018	0.90
Means for driving ewes into the animal milking positions (yells, yes / no)	−0.138	0.33
Means for driving ewes into the animal milking positions (total no. of means used for driving ewes, no.)	0.052	0.71
People participating in driving ewes into the animal milking positions (no.)	−0.245	0.08
Number of working milkers (no.)	−0.143	0.35
Use of gloves by milkers (yes / no)	−0.244	0.08
Provision of concentrate feed during milking (yes / no)	0.133	0.35
Temperature in the milking parlour (°C) (median value of three recordings)	0.623	< 0.0001
Relative humidity in the milking parlour (%) (median value of three reconrdings)	−0.584	< 0.0001
Radio playing within the milking parlour (yes / no)	−0.231	0.10
Application of pre-milking stripping of ewes (yes / no)	n/r	n/r
Correct placement of teatcups on the udder (yes / no)	0.107	0.45
Yells from milkers (yes / no)	0.078	0.58
Early teatcup detachment (yes / no)	−0.207	0.14
End-of-milking stripping of ewes (yes / no)	0.168	0.23
Repeat milking of ewes (yes / no)	−0.110	0.44
Post-milking teat disinfection (yes / no)	−0.280	0.045
Delayed animal exit (yes / no)	−0.006	0.97
Period of preparation for milking process in each milking row	−0.194	0.17
Period of milking for first ewe in each milking row	0.157	0.26
Duration of milking process in each milking row	0.233	0.10
Duration of post-milking actions in each milking row	−0.220	0.88
Duration of milking session in the farm	0.0082	0.56

Table S14.

Results of univariable analysis for potential associations between the independent variables and the outcome total number of distinct reactions observed on ewes on a farm during the milking session.

**Independent variables (*n* = 48)**	r_{sp}	*p*
Number of ewes on the farm (no.)	−0.142	0.32
Breed of ewes (description)	0.115	0.42
Milking session (morning / evening)	−0.160	0.26
Age of farmer (years)	0.157	0.26
Length of previous animal farming experience (years)	0.098	0.49
Level of general education of farmer (primary, secondary, tertiary, professional)	−0.078	0.57
Year of initial establishment (year)	0.111	0.43
Availability of a waiting area before the milking parlour (yes / no)	−0.044	0.76
Availability of ventilators (yes / no)	−0.103	0.47
Type of milking parlour (circular / parallel / linear / other)	−0.217	0.12
Location of animal milking positions in relation to work area of milkers (animals on ramp and milkers on ground / animals on ground and milkers into a pit)	−0.065	0.65
Surface of animal milking positions (m^2)	0.255	0.07
Material of surface of animal milking positions (cement / tile / soil / other)	0.017	0.90
Smoothness of surface of animal milking positions (yes / no)	−0.118	0.40
Number of milking units per animal milking position in the parlour (ratio)	0.207	0.14
Teatcup material (description)	0.201	0.15
System pulsation rate (p. min^{-1})	0.054	0.70
System pressure (kPa)	0.040	0.78
Type of flow line (low / high)	−0.261	0.06
Availability of facilities for milk yield measurement (yes / no)	0.048	0.74
Availability of automatic removal of clusters (yes / no)	n/r	n/r
Frequency of replacement of milking clusters (no. of occasions annually)	0.135	0.34
Ewe congestion before entry into the milking parlour (yes / no)	−0.150	0.29
Means for driving ewes into the animal milking positions (feed, yes / no)	−0.088	0.54
Means for driving ewes into the animal milking positions (whistle, yes / no)	0.110	0.44
Means for driving ewes into the animal milking positions (flexible twig, yes/ no)	0.104	0.46
Means for driving ewes into the animal milking positions (yells, yes / no)	0.111	0.43
Means for driving ewes into the animal milking positions (total no. of means used for driving ewes, no.)	0.128	0.37
People participating in driving ewes into the animal milking positions (no.)	−0.364	0.008
Number of working milkers (no.)	−0.092	0.52
Use of gloves by milkers (yes / no)	−0.306	0.027
Provision of concentrate feed during milking (yes / no)	0.085	0.55
Temperature in the milking parlour (°C) (median value of three recordings)	−0.127	0.37
Relative humidity in the milking parlour (%) (median value of three reconrdings)	0.045	0.75
Radio playing within the milking parlour (yes / no)	0.079	0.58
Application of pre-milking stripping of ewes (yes / no)	n/r	n/r
Correct placement of teatcups on the udder (yes / no)	−0.021	0.88
Yells from milkers (yes / no)	0.124	0.38
Early teatcup detachment (yes / no)	0.162	0.25
End-of-milking stripping of ewes (yes / no)	−0.002	0.99
Repeat milking of ewes (yes / no)	−0.024	0.87
Post-milking teat disinfection (yes / no)	0.143	0.31
Delayed animal exit (yes / no)	−0.142	0.32
Period of preparation for milking process in each milking row	0.039	0.78
Period of milking for first ewe in each milking row	0.232	0.10
Duration of milking process in each milking row	0.067	0.64
Duration of post-milking actions in each milking row	0.011	0.94
Duration of milking session in the farm	0.146	0.30

Figure S1. Scree-plot for principal component analysis for the proportion of ewes that attempted to remove the milking cluster (or removed it) during the milking process (dashed lines are trendlines).

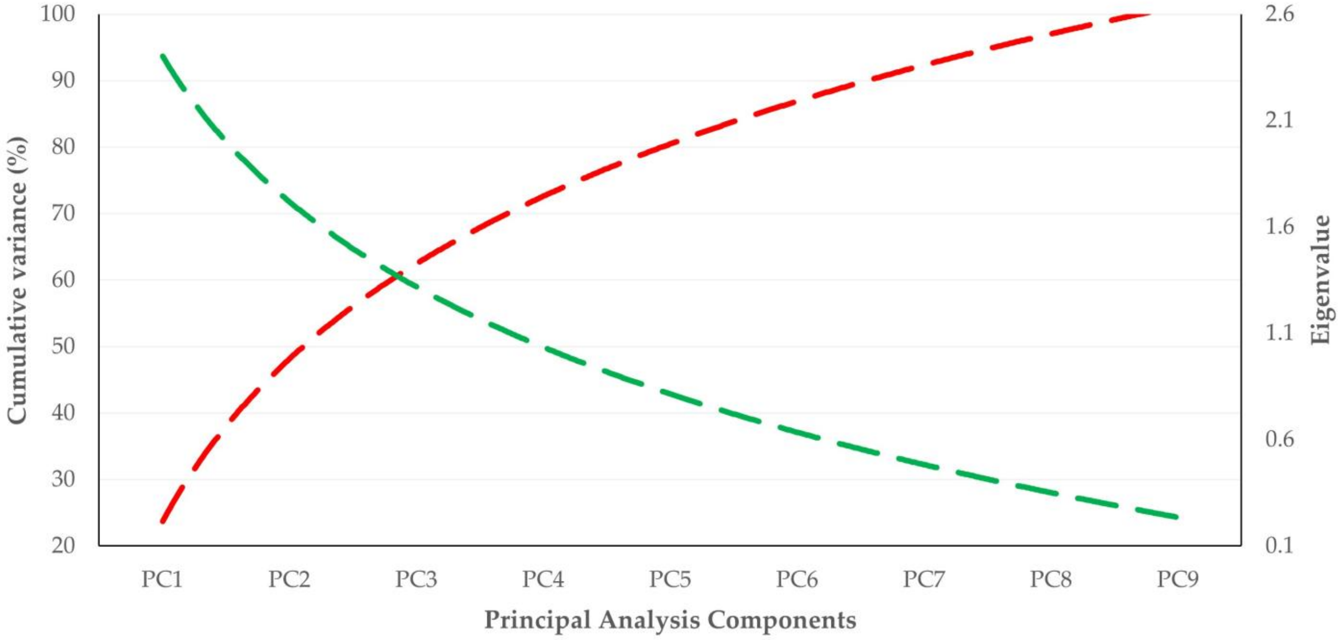

Table S15.

Results of univariable analysis for potential associations between the independent variables and the somatic cell counts in the bulk-tank milk.

Independent variables (*n* = 48)	r_{sp}	*p*
Number of ewes on the farm (no.)	−0.004	0.98
Breed of ewes (description)	−0.094	0.51
Milking session (morning / evening)	0.111	0.43
Age of farmer (years)	0.062	0.66
Length of previous animal farming experience (years)	−0.119	0.40
Level of general education of farmer (primary, secondary, tertiary, professional)	−0.181	0.19
Year of initial establishment (year)	0.001	0.99
Availability of a waiting area before the milking parlour (yes / no)	−0.049	0.73
Availability of ventilators (yes / no)	−0.069	0.63
Type of milking parlour (circular / parallel / linear / other)	−0.150	0.29
Location of animal milking positions in relation to work area of milkers (animals on ramp and milkers on ground / animals on ground and milkers into a pit)	−0.035	0.81
Surface of animal milking positions (m^2)	0.167	0.24
Material of surface of animal milking positions (cement / tile / soil / other)	0.146	0.31
Smoothness of surface of animal milking positions (yes / no)	−0.050	0.72
Number of milking units per animal milking position in the parlour (ratio)	0.125	0.38
Teatcup material (description)	−0.070	0.62
System pulsation rate (p. min^{-1})	−0.084	0.55
System pressure (kPa)	0.014	0.92
Type of flow line (low / high)	0.144	0.31
Availability of facilities for milk yield measurement (yes / no)	−0.250	0.07
Frequency of replacement of milking clusters (no. of occasions annually)	−0.005	0.97
Availability of automatic removal of clusters (yes / no)	n/r	n/r
Ewe congestion before entry into the milking parlour (yes / no)	0.312	0.024
Means for driving ewes into the animal milking positions (feed, yes / no)	−0.052	0.71
Means for driving ewes into the animal milking positions (whistle, yes / no)	0.17	0.23
Means for driving ewes into the animal milking positions (flexible twig, yes/ no)	0.209	0.14
Means for driving ewes into the animal milking positions (yells, yes / no)	−0.158	0.26
Means for driving ewes into the animal milking positions (total no. of means used for driving ewes, no.)	0.148	0.30
People participating in driving ewes into the animal milking positions (no.)	−0.188	0.18
Number of working milkers (no.)	−0.084	0.55
Use of gloves by milkers (yes / no)	−0.146	0.30
Provision of concentrate feed during milking (yes / no)	0.053	0.70
Temperature in the milking parlour (°C) (median value of three recordings)	0.106	0.45
Relative humidity in the milking parlour (%) (median value of three reconrdings)	−0.080	0.57
Radio playing within the milking parlour (yes / no)	−0.019	0.89
Application of pre-milking stripping of ewes (yes / no)	n/r	n/r
Correct placement of teatcups on the udder (yes / no)	0.099	0.49
Yells from milkers (yes / no)	−0.128	0.37
Early teatcup detachment (yes / no)	−0.024	0.87
End-of-milking stripping of ewes (yes / no)	0.092	0.52
Repeat milking of ewes (yes / no)	0.291	0.036
Post-milking teat disinfection (yes / no)	−0.163	0.25
Delayed animal exit (yes / no)	−0.173	0.22
Period of preparation for milking process in each milking row	0.068	0.63
Period of milking for first ewe in each milking row	−0.073	0.61
Duration of milking process in each milking row	0.142	0.32
Duration of post-milking actions in each milking row	−0.112	0.43
Duration of milking session in the farm	−0.143	0.31

Table S16.

Results of univariable analysis for potential associations between the independent variables and the total bacterial counts in the bulk-tank milk.

Independent variables (*n* = 48)	r_{sp}	*p*
Number of ewes on the farm (no.)	−0.031	0.83
Breed of ewes (description)	0.227	0.11
Milking session (morning / evening)	−0.130	0.36
Age of farmer (years)	0.044	0.76
Length of previous animal farming experience (years)	−0.140	0.32
Level of general education of farmer (primary, secondary, tertiary, professional)	−0.049	0.73
Year of initial establishment (year)	0.328	0.018
Availability of a waiting area before the milking parlour (yes / no)	−0.166	0.24
Availability of ventilators (yes / no)	0.132	0.35
Type of milking parlour (circular / parallel / linear / other)	0.057	0.69
Location of animal milking positions in relation to work area of milkers (animals on ramp and milkers on ground / animals on ground and milkers into a pit)	0.268	0.05
Surface of animal milking positions (m^2)	−0.094	0.51
Material of surface of animal milking positions (cement / tile / soil / other)	0.273	0.05
Smoothness of surface of animal milking positions (yes / no)	−0.112	0.43
Number of milking units per animal milking position in the parlour (ratio)	−0.203	0.14
Teatcup material (description)	−0.163	0.25
System pulsation rate (p. min^{-1})	0.056	0.69
System pressure (kPa)	0.173	0.22
Type of flow line (low / high)	0.187	0.18
Availability of facilities for milk yield measurement (yes / no)	0.289	0.038
Availability of automatic removal of clusters (yes / no)	n/r	n/r
Frequency of replacement of milking clusters (no. of occasions annually)	0.035	0.81
Ewe congestion before entry into the milking parlour (yes / no)	0.031	0.83
Means for driving ewes into the animal milking positions (feed, yes / no)	0.114	0.42
Means for driving ewes into the animal milking positions (whistle, yes / no)	−0.052	0.71
Means for driving ewes into the animal milking positions (flexible twig, yes/ no)	0.096	0.50
Means for driving ewes into the animal milking positions (yells, yes / no)	−0.036	0.80
Means for driving ewes into the animal milking positions (total no. of means used for driving ewes, no.)	0.108	0.45
People participating in driving ewes into the animal milking positions (no.)	0.147	0.30
Number of working milkers (no.)	−0.203	0.15
Use of gloves by milkers (yes / no)	0.044	0.76
Provision of concentrate feed during milking (yes / no)	0.267	0.06
Temperature in the milking parlour (^{o}C) (median value of three recordings)	0.182	0.19
Relative humidity in the milking parlour (%) (median value of three reconrdings)	−0.049	0.73
Radio playing within the milking parlour (yes / no)	−0.058	0.68
Application of pre-milking stripping of ewes (yes / no)	n/r	n/r
Correct placement of teatcups on the udder (yes / no)	−0.122	0.39
Yells from milkers (yes / no)	0.087	0.54
Early teatcup detachment (yes / no)	−0.007	0.96
End-of-milking stripping of ewes (yes / no)	0.021	0.88
Repeat milking of ewes (yes / no)	0.123	0.39
Post-milking teat disinfection (yes / no)	−0.288	0.038
Delayed animal exit (yes / no)	0.023	0.87
Period of preparation for milking process in each milking row	0.192	0.17
Period of milking for first ewe in each milking row	0.152	0.28
Duration of milking process in each milking row	0.153	0.28
Duration of post-milking actions in each milking row	0.164	0.25
Duration of milking session in the farm	−0.016	0.91

Table S17.

Results of correlation analyses for potential associations between the various reactions observed in ewes during the milking process and the characteristics of bulk-tank milk produced on the farms.

Outcomes (n = 9)	Somatic cell counts		Total bacterial counts	
	r_{sp}	p	r_{sp}	p
% of ewes that displayed 'Kneeling' before entry to the milking pen	−0.083	0.55	0.154	0.28
% of ewes that displayed 'Kneeling' within the milking pen	0.172	0.22	0.157	0.27
% of ewes that urinated	0.151	0.29	−0.181	0.20
% of ewes that defaecated	−0.181	0.20	0.012	0.93
Vocalisation	0.037	0.79	−0.080	0.57
% of ewes that displayed kick-like reaction	0.161	0.25	0.087	0.54
% of ewes that attempted to remove the milking cluster (or removed it)	0.315	0.023	−0.074	0.60
% of ewes that showed spot stepping	0.187	0.18	−0.036	0.81
total number of distinct reactions observed on ewes on a farm during the milking session	0.120	0.40	−0.020	0.89
